# Gas Separation by Sorption: A Statistical Thermodynamic Fluctuation Theory

**DOI:** 10.1021/acs.langmuir.6c02601

**Published:** 2026-08-31

**Authors:** Seishi Shimizu, Nobuyuki Matubayasi

**Affiliations:** † York Structural Biology Laboratory, Department of Chemistry, 8748 University of York, Heslington, York YO10 5DD, U.K.; ‡ Division of Chemical Engineering, Graduate School of Engineering Science, 13013 Osaka University, Toyonaka, Osaka 560-8531, Japan

## Abstract

To understand how gas mixtures can be separated and purified by adsorption, we present a novel theory of selective sorption that (i) is applicable to any interfacial geometry and porosity, (ii) synthesizes sorption and activity with built-in thermodynamic consistency, and (iii) incorporates the inhomogeneous distribution of sorbates at the interface. Number correlations from the fluctuation theory provide a unified quantification of sorbate–surface and sorbate–sorbate interactions in the language common to the liquid theory. From the gradient of the isotherm alone, our theory can reveal the sorbate interactions that underlie selective sorption. The multicomponent ABC isotherm, derived from our theory, can successfully model gas-mixture adsorption without the need for implicit functions to maintain thermodynamic consistency between activity and adsorption. Our AB isotherm captures sorption selectivity while retaining the mathematical simplicity of the Extended Langmuir model. Handling attractive and repulsive interactions between sorbates was shown to be crucial, which was beyond the reach of previous isotherm models.

## Graphical abstract

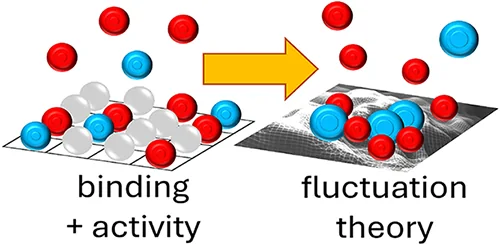

## Introduction

Adsorbents^1,2^ and polymer membranes^3–5^ are commonly used for gas separation and purification. The key to these two processes is selective sorption, which can be quantified by multicomponent sorption isotherms (i.e., measuring the adsorption of each gas component while varying partial pressures).^6–9^

Given a multicomponent sorption isotherm, how can we gain insight into the mechanism of selective sorption? In the following, we will summarize (i) what the previous approaches aimed to capture, (ii) what the essential processes are for selective sorption, and (iii) how all the essential processes in (ii) can be encompassed naturally in a unified theory without employing ad-hoc models and assumptions.

### Adsorption-Activity Dichotomy in Modeling Selective Sorption

#### Adsorption versus Activity

Previous approaches to modeling selective sorption can largely be classified into three categories: (i) adsorption models (Figure 1a),^17^ (ii) activity models (Figure 1b),^15,18^ and (iii) the synthesis of (i) and (ii) in a thermodynamically consistent way (Figure 2).^15,18^ The first approach to selective sorption is extending the Langmuir model, originally proposed for pure gas isotherms^19,20^ to multicomponent sorbate mixtures.^10–12^ This Extended Langmuir model (Figure 1a) employs the adsorption sites and binding constants for each adsorbate.^9,17,21^ However, the Extended Langmuir model cannot (1) fulfill consistency with the Gibbs isotherm for adsorbates with different saturation capacities,^16,22^ (2) account for adsorbate size differences,^18,23^ and (3) handle adsorption preference reversal between low and high pressures.^18,23^ Attempts have been made to overcome such limitations of the Extended Langmuir Model to homogeneous adsorbate mixtures^9,16,24^ via the addition of the Henry term.^25,26^ This leads us to the second category, the activity models, which aim to provide direct insight into the adsorbed phase. The ideal adsorbed solution theory (IAST) assumes the ideal mixing of adsorbates as the simplest possible scenario for (1).^27–29^ The real adsorbed solution theory (RAST) achieved improvement over IAST by incorporating the activity coefficients.^16,30–33^ Sorbate size difference was accounted for by the Flory–Huggins model, while simplifying larger sorbates as connected sites in a lattice (cell)^34,35^ and the open (unadsorbed) surface patches (Figure 1b) were incorporated via “vacancy” ^13,14^ or “phantom molecules”.^15,16^ Furthermore, the nonrandom two-liquid (NRTL) model,^36^ which has been adopted for adsorbed phase nonideality,^37–39^ has addressed the need for “local” concentration inhomogeneity around each sorbate species (Figure 3a). Despite these successes, an activity model (which, by definition, predicts gas adsorption from partial gas pressures or activities) is an inverse function of a sorption isotherm.^40,41^ This is a major drawback because selective adsorption is commonly measured as a function of partial gas pressures.^42–44^

**Figure 1:**
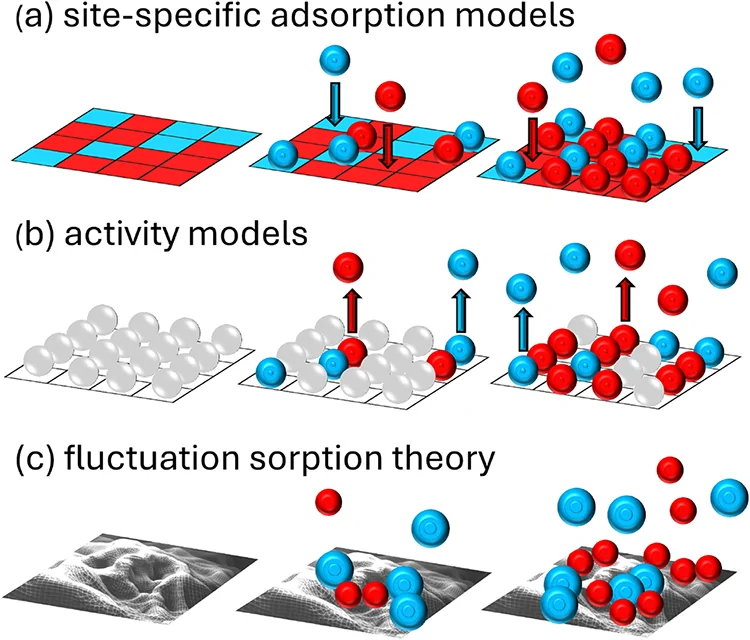
Different approaches to model selective sorption of gas mixtures (represented by blue and red spheres). (a) Adsorption isotherm models, such as the Extended Langmuir model,^10–12^ describe local (site-specific) adsorption on homogeneous surfaces. (b) Activity models for the mixture (blue and red spheres), with the incorporation of unoccupied sites via “vacancy” or “phantom molecules” (gray spheres),^13–16^ predict the relative pressure of each species as a function of surface adsorbate concentration; (c) Our fluctuation sorption theory is applicable to any interfacial geometry (shown schematically) and to localized and mobile adsorptions alike.

**Figure 2:**
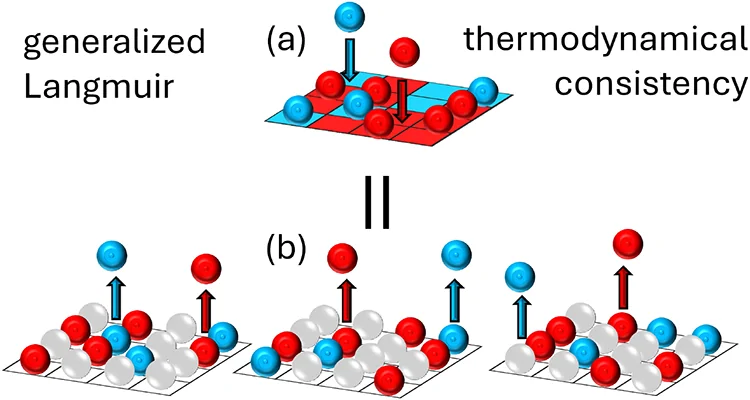
Generalized Langmuir model as an attempt to synthesize (a) site-specific adsorption (adsorbate concentrations as a function of partial pressures) and (b) surface activity (local pressures as functions of concentrations) in a thermodynamically consistent manner.^9,15,16^ The multicomponent extension of the Langmuir model via activity is used for (a)^9,17,21^ and the NRTL model^9,36^ is used for (b). Throughout this paper, species 1 and 2 are represented by blue and red, respectively.

**Figure 3:**
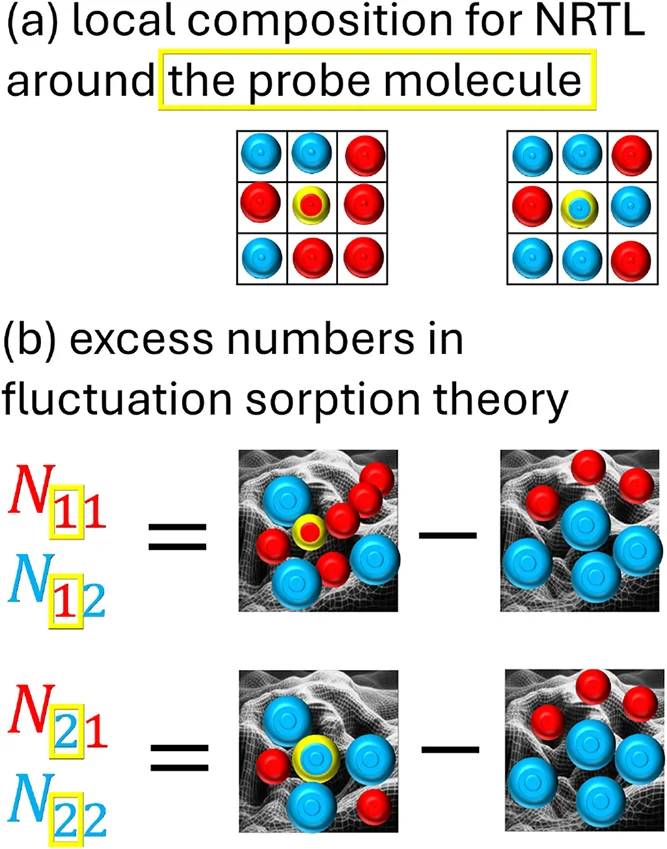
Incorporating sorbate–sorbate interactions at the interface. (a) NRTL via local concentration around the probe sorbate molecule with a yellow surface.^36–39^ (b) Fluctuation sorption theory in this paper, via *N*_*ij*_, i.e., the excess number of species *j* around the probe *i* with a yellow surface, as the normalized number correlation.

#### Synthesizing Adsorption and Activity Models

The adsorption and activity models were synthesized to overcome their respective weaknesses identified in the previous paragraph. The Generalized Langmuir model (Figure 2) was proposed as a synthesis of the multicomponent Langmuir model for adsorption and the NRTL model for activity.^9,15,16^ Its success can be attributed to the thermodynamic consistency ensured between the adsorption and activity.^9,15,16^ However, two limitations remain unresolved in the Generalized Langmuir approach. The first is algorithmic complications arising from thermodynamic consistency: solving simultaneous equations involving mutually inverse functions (adsorption and activity models)^9,15,16^ necessitates handling implicit functions. The second is the inability to incorporate local concentration beyond the immediate vicinity of the probe due to the simplified model assumption of the NRTL model (Figure 3a; see the next paragraph for details). These two limitations will be overcome by the fluctuation sorption theory (Figure 1c).

### Selective Sorption via Interfacial Sorbate Distributions

#### Local Concentrations Beyond the Immediate Vicinity

The NRTL model (Figure 3a), which has been adopted for the Generalized Langmuir model, is capable of capturing local concentrations, i.e., the difference in the composition of the solution between the locality (vicinity) of a species and the bulk. The local concentration was originally introduced as a correction factor to improve the prediction of solution activity by the lattice (cell) models within mean-field approximation (e.g., Flory–Huggins).^45–47^ Such local solution inhomogeneities can be captured by the fluctuation theory, using particle number correlations between every pair of molecular species (Figure 3b) without any assumptions. Indeed, number correlations are the foundations for the exact theories of solutions (McMillan-Mayer^40^ and Kirkwood-Buff^48–52^ ). Thus, the number correlations (Figure 3b) are a natural and model-free generalization of the local concentration model (Figure 3a); both aim to capture molecular distributions around a probe molecule. We emphasize the key difference between the two: while NRTL is based on the lattice model and focuses on local vicinities (Figure 3a), the fluctuation theory is free of model assumptions and approximations, hence it is applicable to any interfacial geometry, sorbate size, and shape (Figure 3b).

#### Extending Fluctuation Sorption Theory to Mixtures

Quantifying sorbate interactions via number correlations has a proven track record of overcoming long-standing difficulties in sorption modeling.^41,53,54^ First, the ABC isotherm has replaced the site-specific and/or layer-by-layer adsorption models, such as the Langmuir,^19,20^ BET,^55^ and GAB,^56–58^ providing universal mechanistic interpretation via sorbate-surface, disorbate, and trisorbate interactions.^41,53,54^ (It has also generalized the classical models^59^ for sorption from solution to the fluctuation theory-based isotherms.^60^) Second, the isotherms derived from the fluctuation theory can be applied to any interfacial shape or porosity, for “localized” and “mobile” modes of adsorption,^58^ both on “homogeneous” and “inhomogeneous” types of interfaces.^41,54,61^ Third, our approach is free of complications to isotherm data analysis arising from inverse functions (“relative pressure (activity) as a function of adsorbed quantity” rather than “adsorbed quantity as a function of relative pressure (activity)”) as an inevitable consequence of employing the interfacial equation of state (EOS),^17,43,58,62,63^ which nevertheless failed to generalize the Langmuir, BET, and GAB models beyond their idealized, site-specific assumptions.^41,61^ Based on this track record, this paper will generalize the fluctuation sorption theory from single-component (unary)^41,53,54^ to multicomponent sorbates.

### Aims and Objectives

Our goal is to elucidate gas separation via sorption on a mechanistic basis. This requires quantifying the interactions underlying the selective and competitive sorption of gases. Our aim is to establish how such sorbate interactions can be evaluated from experimental sorption isotherms. This requires us to generalize our statistical thermodynamic fluctuation theory for pure sorbates^41,53,54,61,64,65^ to sorbate mixtures. To this end, the present paper will construct the following theoretical tools founded on our generalized Gibbs isotherm:^41,61,64^

1. the fundamental relationships for competitive sorption;
2. the ABC sorption isotherm for sorbate mixtures;
3. (1) and (2) specifically adapted for sorption selectivity measurements along constant total pressure;

Based on these theoretical tools, our objectives are

I. to reveal the mechanism of selective sorption directly from experimental data without any model assumptions;
II. to quantify sorbate interactions underlying selective sorption via the ABC isotherm for binary sorbate mixtures;
III. to demonstrate that both sorbate–sorbate attraction and repulsion at the interface must be incorporated for modeling binary sorption isotherms.

Through these objectives, we will identify the unequal treatment of attraction and repulsion as the key limitations of the classical isotherm models that our new approach will overcome. Thus, our focus is on how to interpret sorption isotherms mechanistically, directly from the fluctuation theory, instead of proposing, at the present stage, a predictive scheme.

## Theory

Our presentation will focus on binary mixtures for simplicity, yet it can be generalized straightforwardly to multicomponent mixtures. In doing so, we will generalize our derivation of single sorbate (unary) isotherms, directly from the statistical thermodynamic fluctuation theory, presented in detail in our previous papers (see eq 7–10 of ref 41). As a notation, we adopt species *i* = *e* as sorbent and *i* = 1 and 2 as sorbates (in accordance with the practice in the literature). For “surface”, we use the subscript *s*. The superscript *b* denotes the bulk reference phase. Note that our theory applies to adsorption and absorption, hence we will employ the term “sorbate” throughout.

### Model-Free Interpretation of Competitive Sorption (Tool 1)

#### Generalized Gibbs Isotherm

Our goal is to establish a theoretical basis for a binary sorption isotherm. To guarantee the applicability of our theory to arbitrary interfacial geometry and porosity, as well as to any mode of sorption, our foundation is the generalized Gibbs isotherm,^64^ which introduces the dividing surface purely algebraically via Legendre transformation of statistical ensembles (see Supporting Information: A).^53,64–66^ This approach is advantageous over the coordinate-based introduction of the dividing surface by Gibbs,^67^ which is difficult to define for porous, complex, and heterogeneous surfaces.^53,64–66^ Thus, due to the generality of our theoretical foundation, our **Tool 1** applies to any experiments and simulations.

#### Binary Sorption Isotherm

Based on the generalized Gibbs isotherm, first we introduce a 2-dimensional vector of the surface excess of species *i*, Γ_*i*_,

1 $$\Gamma \equiv \left(\Gamma_{1},\Gamma_{2}\right)$$

where

2 $$\Gamma_{i}=\left\langle n_{i}\right\rangle -\left\langle n_{i}^{s}\right\rangle -\left\langle n_{i}^{b}\right\rangle$$

is a difference in ensemble-averaged numbers (⟨ ⟩) between (i) species *i* at the interface (*n*_*i*_) and (ii) in the bulk reference systems of the solid adsorbent (*n*_*i*_^*s*^) and the bulk gas (*n*_*i*_^*b*^) sides. Note that *n*_*i*_, *n*_*i*_^*s*^, *n*_*i*_^*b*^ are confined within the volume *v* that contains all interfacial effects.^61,64–66^ Second, the independent variable is the 2-dimensional vector **P** = (*P*_1_, *P*_2_), where *P*_*i*_ is the partial pressure of species *i* in the bulk. However, instead of **P**, we will introduce the density vector defined as

3 $$\rho \equiv \left(\rho_{1},\rho_{2}\right),,\rho_{i}=\frac{P_{i}}{\mathrm{RT}}$$

Note that ρ_*i*_ = ⟨*n*_*i*_^*b*^⟩/*v* with the ideal gas equation of state. Adopting **ρ** has three advantages: (i) a clearer presentation of the theory (as will be demonstrated in the following), (ii) the use of the ideal gas density for ρ_*i*_, and (iii) applicability to sorbate gases above the critical temperature at which the saturation pressure cannot be defined. Thus, we will adopt **ρ** as the independent variable such that the binary isotherm is defined as

4 Γ=Γ(ρ)

Our objective is to determine the functional form of **Γ** = **Γ**(**ρ**) based directly on the fundamental principles of fluctuation theory.

#### Logarithmic Isotherm Gradient

Having defined a binary sorption isotherm, here we establish the fundamental relationship for the gradient of a binary sorption isotherm. In doing so, our theoretical foundation is our ensemble-based generalization of the Gibbs isotherm, applicable to any interfacial geometry and porosity.^61,64^ Its straightforward extension to sorbate mixtures is summarized in Supporting Information: A. Focusing on the amount of sorption, i.e., Γ_*i*_ ≃ ⟨*n*_*i*_⟩, the logarithmic gradient of the sorption vector component obeys

5 $${\left(\frac{\partial \;\ln\left\langle n_{i}\right\rangle }{\partial \;\ln\rho_{j}}\right)}_{T}=N_{\mathrm{ij}}+\delta_{\mathrm{ij}}$$

where *N*_*ij*_ is the excess number of species *j* around a probe molecule of species *i*, fixed at the origin in the interfacial subsystem (see Figure 3b), and δ_*ij*_ is Kronecker’s delta (Supporting Information: A). There are two equivalent ways of interpreting the interfacial excess number (*N*_*ij*_), as^41,61^

6 $$N_{\mathrm{ij}}=\frac{\left\langle \delta n_{i}\delta n_{j}\right\rangle }{\left\langle n_{i}\right\rangle }-\delta_{\mathrm{ij}}={\left\langle n_{j}\right\rangle }_{i}-\left\langle n_{j}\right\rangle$$

The first is through the number correlation ⟨*δn*_*i*_ *δn*_*j*_⟩ with *δn*_*i*_ = *n*_*i*_ – ⟨*n*_*i*_⟩ as the deviation from the mean. The second is the difference in the ensemble average of *n*_*j*_ between the presence (⟨*n*_*j*_⟩_*i*_, referred to as the inhomogeneous ensemble^68,69^ ) and absence (⟨*n*_*j*_⟩) of the probe molecule of species *i* fixed at the origin (as has been represented schematically in Figure 3b). Both expressions clarify the role of *N*_*ij*_ as a measure of interactions: a positive *N*_*ij*_ indicates the excess of species *j* located around the probe (species *i*), while a negative *N*_*ij*_ denotes the deficit.^41,61^ Both expressions will be useful in establishing a physical interpretation of the isotherm parameters. Thus, through eq 5, the logarithmic (ln ρ_*j*_) gradient of an isotherm component ln ⟨*n*_*i*_⟩ has been linked to *N*_*ij*_ as the measure of overall sorbate–sorbate interactions^64,65^ that encompasses attraction and repulsion. This link is valid for any interfacial geometry and porosity, as well as for any sorbate molecules.

#### Selectivity Coefficient

In the experimental literature, a common quantitative measure of the selective sorption of sorbate *i* over sorbate *j* is the selectivity coefficient (or separation factor),^9,33^ defined as

7 $$S_{i/j}\equiv \frac{\left\langle n_{i}\right\rangle }{\left\langle n_{j}\right\rangle }\frac{\rho_{j}}{\rho_{i}}$$

which also signifies the equilibrium coefficient for exchanging “*i* at the interface and *j* in the bulk” with “*j* at the interface and *i* in the bulk”. Focusing on *S*_1/2_, how it changes with the gas partial pressures yields mechanistic insight into sorbate interactions at the interface, via

8 $$d\;\ln S_{1/2}=\left(N_{11}-N_{21}\right)d\;\ln\rho_{1}+\left(N_{12}-N_{22}\right)d\;\ln\rho_{2}$$

which can be derived straightforwardly by combining eqs 7 and 5. An immediate consequence of eq 8 is

9 $$\begin{array}{l} N_{11}-N_{21}={\left(\frac{\partial \;\ln{\;S}_{1/2}}{\partial \;\ln\rho_{1}}\right)}_{\rho_{2}}\\ N_{12}-N_{22}={\left(\frac{\partial \;\ln{\;S}_{1/2}}{\partial \;\ln\rho_{2}}\right)}_{\rho_{1}} \end{array}$$

In light of eq 6, *N*_11_ – *N*_21_ of eq 9 signifies the preference of species 1 to interact with probe sorbate 1 over probe sorbate 2. This can be evaluated by experimentally plotting ln *S*_1/2_ against ln ρ_1_ while keeping ρ_2_ constant. Likewise, *N*_12_ – *N*_22_ can be evaluated similarly from the ln ρ_2_ dependence of ln *S*_1/2_ via eq 9. Thus, the fluctuation theory can reveal preferential sorbate interactions at the interface solely from the selectivity coefficient without any model assumptions.

#### Surface-Sorbate Interactions

With the sorption isotherm (⟨*n*_1_⟩,⟨*n*_2_⟩), we can quantify all the four excess numbers (*N*_11_, *N*_12_, *N*_21_, and *N*_22_) underlying preferential sorbate interactions (*N*_11_ – *N*_21_ and *N*_12_ – *N*_22_) individually without any model assumptions. Here, our goal is twofold: to connect the selectivity coefficient to the surface-sorbate KBI, *G*_*si*_, which is the universal measure of surface-sorbate interaction, and to establish a link between *G*_*si*_ and the excess numbers. The selectivity coefficient is the competitive sorption of species 1 and 2,

10 $$d\;\ln S_{1/2}=d\;\ln\frac{\left\langle n_{1}\right\rangle }{\rho_{1}}-d\;\ln\frac{\left\langle n_{2}\right\rangle }{\rho_{2}}$$

as can be derived straightforwardly from eq 7, each linked to the excess numbers, via

11 $$\begin{array}{l} d\;\ln\frac{\left\langle n_{1}\right\rangle }{\rho_{1}}=N_{11}d\;\ln\rho_{1}+N_{12}d\;\ln\rho_{2}≃d\;\ln G_{s1}\\ d\;\ln\frac{\left\langle n_{2}\right\rangle }{\rho_{2}}=N_{21}d\;\ln\rho_{1}+N_{22}d\;\ln\rho_{2}≃d\;\ln G_{s2} \end{array}$$

that result from eq 5 straightaway. Here, the link to the surface-sorbate KBI in eq 11 can be established via its definition

12 $$G_{\mathrm{si}}\equiv v\frac{\left\langle n_{i}\right\rangle -\left\langle n_{i}^{b}\right\rangle }{\left\langle n_{i}^{b}\right\rangle }≃\frac{\left\langle n_{i}\right\rangle }{\rho_{i}}$$

where the approximation holds at low pressures (Γ_*i*_ ≃ ⟨*n*_*i*_⟩) and under the ideal gas equation of state (ρ_*i*_ = ⟨*n*_*i*_^*b*^ ⟩/*v*). From eq 11, all four excess numbers can be evaluated from experimental isotherms alone, via

$$\begin{array}{l} N_{11}={\left(\frac{\partial \ln{\;G}_{s1}}{\partial \ln\rho_{1}}\right)}_{\rho_{2}},,N_{12}={\left(\frac{\partial \ln{\;G}_{s1}}{\partial \ln\rho_{2}}\right)}_{\rho_{1}}\\ N_{21}={\left(\frac{\partial \ln G_{s2}}{\partial \ln\rho_{1}}\right)}_{\rho_{2}},,N_{22}={\left(\frac{\partial \ln{\;G}_{s2}}{\partial \ln\rho_{2}}\right)}_{\rho_{1}} \end{array}$$ 13

Thus, we have established how all four interfacial sorbate–sorbate excess numbers can be determined from experimental isotherm data.

To summarize, from the experimental isotherm alone, surface-sorbate and sorbate–sorbate interactions can be quantified in terms of their number correlations that share the same language as the molecular distributions calculated by simulation.^41^

### The ABC Isotherm for Binary Mixtures (Tool 2)

#### Bridging Macroscopic Isotherms to Microscopic Interactions

Our goal is to fill the long-standing gap between computer simulation (e.g., molecular dynamics and classical density functional theory) and experimental isotherm analysis.^41^ While computer simulation can directly evaluate molecular distributions, the current isotherm models (e.g., Langmuir, BET, and GAB and their multicomponent generalizations), that are used to fit macroscopic isotherm data, are founded on primitive models (e.g., cell or lattice models and site-specific stoichiometric binding).^41^ Our generalization of the ABC isotherm, which will be presented below, aims to bridge the two, by reformulating the isotherm based on sorbate fluctuation, with a direct link to the molecular distribution functions.^41,53,54,64,65^

#### Isotherm-Generating Relationship

Here, we will derive the ABC isotherm for binary mixtures by generalizing our method for deriving isotherm equations for unary sorbates.^41,54,61^ (See Supporting Information: B for a summary on how the unary ABC isotherm can be derived, how it provides a unified mechanistic explanation for IUPAC Types I–III, how it can be generalized naturally to liquid isotherms, and how the fluctuation theory explains the temperature dependence of sorption. Although this subsection covers the full (ρ_1_, ρ_2_) landscape, we shall demonstrate in the next subsection that we can still extract key mechanistic insights from a combination of pure gas isotherms and isotherms along constant total pressure.) The first step is to derive the isotherm-generating relationship, which can be achieved simply by rewriting eq 11 into the following form:

14 $$\begin{array}{l} -d\frac{\rho_{1}}{\left\langle n_{1}\right\rangle }=\frac{G_{11}}{v}d\rho_{1}+S_{2/1}\frac{G_{12}}{v}d\rho_{2}\\ -d\frac{\rho_{2}}{\left\langle n_{2}\right\rangle }=S_{1/2}\frac{G_{21}}{v}d\rho_{1}+\frac{G_{22}}{v}d\rho_{2} \end{array}$$

where the sorbate–sorbate interfacial KBIs are defined as

15 $$G_{\mathrm{ij}}=\frac{v}{\left\langle n_{j}\right\rangle }N_{\mathrm{ij}}$$

which serves as an alternative measure for sorbate–sorbate correlation, with a direct link to spatial integration of distribution functions.^41^ Note, by definition (eqs 6 and 15), that *G*_*ij*_ = *G*_*ji*_, whereas *N*_*ij*_ ≠ *N*_*ji*_. We emphasize that the isotherm-generating relationship (eq 14) is an alternative yet mathematically equivalent expression of the exact theory presented in the previous subsection. Not only will it lead naturally to the ABC isotherm as a generalization of Langmuir, BET, and GAB models but also facilitate the physical interpretation of its parameters.

#### Characteristic Equation

The second step to derive an isotherm is to establish a characteristic equation.^41,61,70^ Our aim is to generalize the ABC isotherm for single components. Consequently, we will adopt the following expansion around **ρ** ^*r*^ = (ρ_1_^*r*^, ρ_2_^*r*^), using Δ*ρ*_*i*_ ≡ ρ_*i*_ – ρ_*i*_^*r*^

$$\begin{array}{ccc} \frac{G_{11}}{v} & = & B_{11}^{\rho^{r}}+C_{111}^{\rho^{r}}\Delta \rho_{1}+C_{112}^{\rho^{r}}\Delta \rho_{2}+\cdot \cdot \cdot \\ S_{2/1}\frac{G_{12}}{v} & = & B_{12}^{\rho^{r}}+C_{121}^{\rho^{r}}\Delta \rho_{1}+C_{122}^{\rho^{r}}\Delta \rho_{2}+\cdot \cdot \cdot \\ \frac{G_{22}}{v} & = & B_{22}^{\rho^{r}}+C_{222}^{\rho^{r}}\Delta \rho_{2}+C_{221}^{\rho^{r}}\Delta \rho_{1}+\cdot \cdot \cdot \\ S_{1/2}\frac{G_{21}}{v} & = & B_{21}^{\rho^{r}}+C_{212}^{\rho^{r}}\Delta \rho_{2}+C_{211}^{\rho^{r}}\Delta \rho_{1}+\cdot \cdot \cdot \end{array}$$ 16

as a binary generalization of the ABC characteristic equation. In eq 16, *B*_*ij*_^**ρ** ^*r*^^ and *C*_*ijk*_^**ρ** ^*r*^^ are expansion coefficients defined at **ρ** ^*r*^ = (ρ_1_^*r*^, ρ_2_^*r*^), whose interpretation will be clarified by eqs 19–21, below.

#### ABC Isotherm for Binary Mixtures

The third step to derive the isotherm is to integrate the isotherm generating relationship (eq 14) alongside the characteristic equation (eq 16). Through simple algebra (see Supporting Information: C), we obtain

$$\begin{array}{l} \left\langle n_{1}\right\rangle =\frac{\rho_{1}}{A_{1}^{\rho^{r}}-B_{11}^{\rho^{r}}\Delta \rho_{1}-B_{12}^{\rho^{r}}\Delta \rho_{2}-\frac{C_{111}^{\rho^{r}}}{2}{\left(\Delta \rho_{1}\right)}^{2}-\frac{C_{122}^{\rho^{r}}}{2}{\left(\Delta \rho_{2}\right)}^{2}-C_{112}^{\rho^{r}}\Delta \rho_{1}\Delta \rho_{2}}\\ \left\langle n_{2}\right\rangle =\frac{\rho_{2}}{A_{2}^{\rho^{r}}-B_{21}^{\rho^{r}}\Delta \rho_{1}-B_{22}^{\rho^{r}}\Delta \rho_{2}-\frac{C_{211}^{\rho^{r}}}{2}{\left(\Delta \rho_{1}\right)}^{2}-\frac{C_{222}^{\rho^{r}}}{2}{\left(\Delta \rho_{2}\right)}^{2}-C_{221}^{\rho^{r}}\Delta \rho_{1}\Delta \rho_{2}} \end{array}$$ 17

which is a generalization of the ABC isotherm to binary sorbate mixtures. Indeed, eq 17 contains the ABC isotherm for pure gases (single-component sorbates). This can be demonstrated easily by putting ρ_2_ = 0 and by choosing **ρ** ^*r*^ = (0,0) in eq 17, which yields

18 $$\left\langle n_{1}\right\rangle =\frac{\rho_{1}}{A_{1}^{\left(0,0\right)}-B_{11}^{\left(0,0\right)}\rho_{1}-\frac{1}{2}C_{111}^{\left(0,0\right)}\rho_{1}^{2}}$$

which is the ABC isotherm for pure sorbates that we derived recently.^53,54,65^ This isotherm contains the Langmuir, BET, and GAB isotherms as its special cases.^65^ Table 1 summarizes the correspondence between our isotherm parameters (*A*_1_^(0,0)^, *B*_11_^(0,0)^, and *C*_111_^(0,0)^) and the Langmuir, BET, and GAB parameters, derived in full in ref 65. (For more details on unary isotherms, see Supporting Information: B). We emphasize that our binary ABC isotherm is applicable to any interfacial geometry, even in the presence of absorption, directly from the fundamentals of the fluctuation theory. Just like its unary counterpart (eq 18), it contains the extended Langmuir model as its special case, as will be discussed in the next subsection. Generalization to multicomponent sorbate mixtures can be achieved similarly, yet via tedious algebra.

**Table 1:** Correspondence between the Unary ABC Isotherm Parameters (eq 18) and the Langmuir, BET, and GAB Models^a^

model	*A*_1_^(0,0)^/ρ_1_^o^	*B*_11_^(0,0)^	*C*_111_^(0,0)^ρ_1_^o^
Langmuir	$\frac{1}{n_{m}K_{L}}$	$-\frac{1}{n_{m}}$	0
BET	$\frac{1}{C_{B}n_{m}}$	$\frac{2-C_{B}}{C_{B}n_{m}}$	$\frac{2\left(C_{B}-1\right)}{C_{B}n_{m}}$
GAB	$\frac{1}{C_{B}K_{G}n_{m}}$	$\frac{2-C_{B}}{C_{B}n_{m}}$	$\frac{2K_{G}\left(C_{B}-1\right)}{C_{B}n_{m}}$

a *n*_*m*_: monolayer capacity; *K*_*L*_: Langmuir constant; *C*_*B*_: BET parameter; *K*_*G*_: GAB parameter. BET is the *K*_*G*_ = 1 special case of GAB. Conversion to the activity scale is via ρ_1_ = ρ_1_^o^ *a*_1_, where ρ_1_^o^ is the saturation value of ρ_1_. Full derivation in ref 65.

#### Parameter Interpretations

A clear interpretation of the ABC parameters can be achieved via the fluctuation theory, as will be demonstrated below. First, combining eqs 12 and 17 leads straightforwardly to

19 $$\frac{1}{A_{i}^{r}}={\left(G_{\mathrm{si}}\right)}_{\rho^{r}}$$

as the surface-sorbate KBI at **ρ** = **ρ** ^*r*^. Note that adopting ρ_*i*_ (instead of sorbate activity *a*_*i*_ or partial pressure *P*_*i*_) has a clear advantage in establishing a direct link between 1/*A*_*i*_^*r*^ and *G*_*si*_. Second, *B*_*ij*_^**ρ** ^*r*^^ has a direct link to sorbate–sorbate KBI and the selectivity coefficient, via

20 $$B_{\mathrm{ij}}^{\rho^{r}}={\left(S_{j/i}\frac{G_{\mathrm{ij}}}{v}\right)}_{\rho =\rho^{r}}$$

which is a straightforward consequence of eq 16. Note that *S*_*i*/*i*_ = 1 has been employed throughout our derivations. Interpreting *C*_*ijk*_ requires significant algebra (see Supporting Information: C).

21 $$\begin{array}{l} C_{111}^{\rho^{r}}=\frac{1}{A_{1}^{r}}{\left[\frac{\langle n_{1}\rangle_{1}N_{1,11}-\left\langle n_{1}\right\rangle N_{11}}{\langle n_{1}\rangle^{2}}-B_{11}^{2}\right]}_{\rho =\rho^{r}}\\ C_{112}^{\rho^{r}}=C_{121}^{\rho^{r}}=\frac{1}{A_{2}^{r}}{\left[\frac{\langle n_{1}\rangle_{1}N_{1,12}-\left\langle n_{1}\right\rangle N_{12}-N_{11}N_{12}}{\left\langle n_{1}\right\rangle \left\langle n_{2}\right\rangle }\right]}_{\rho =\rho^{r}}\\ C_{122}^{\rho^{r}}=\frac{A_{1}^{r}}{{\left(A_{2}^{r}\right)}^{2}}{\left[\frac{\langle n_{2}\rangle_{1}N_{1,22}-\left\langle n_{2}\right\rangle N_{22}-N_{12}^{2}}{\langle n_{2}\rangle^{2}}\right]}_{\rho =\rho^{r}} \end{array}$$

Thus, the ABC parameters can be interpreted solely in terms of mean numbers and fluctuations, without any model assumptions about interfacial geometry, porosity, or binding specificity. Consequently, our ABC isotherm is founded on the same theoretical language as molecular dynamics simulation and classical density functional theory that captures the spatial distribution of molecules.^41^ Our ABC isotherm for mixtures (eq 17) encompasses experimental isotherm measurements commonly made along constant total pressure (see below) and constant gas composition.^71–73^

### Selective Sorption Along Constant Total Pressure (Tool 3)

#### Utilizing Limited Experimental Coverage

Even for binary gas mixtures, it is difficult experimentally to determine an isotherm vector (⟨*n*_1_⟩, ⟨*n*_2_⟩) over a wide range of the (*P*_1_, *P*_2_) space. A standard practice is to keep the total pressure constant (*P* = *P*_1_ + *P*_2_ = const, equivalent to ρ = ρ_1_ + ρ_2_ = const), while varying the gas composition along this line (choosing the gas-phase mole-fraction of species *i*, *y*_*i*_, as the variable for binary mixtures).^9,18,30,33,72,73^ Under such limited coverage, how can we still gain mechanistic insights? Significant interpretive clarity, both for model-free and ABC isotherm approaches, results from studying selectivity along constant ρ, as will be demonstrated in the following. To this end, the number of variables can be reduced via

22 $$d\rho_{2}=-d\rho_{1},,d\rho_{1}=\rho dy_{1}$$

We will demonstrate the interpretive facility resulting from eq 22.

#### Model-Free Interpretation

The model-free interpretation of the selectivity coefficient *S*_1/2_ can be facilitated for measurements along a constant total pressure. Under eq 22, eq 8 reduces to

23 $$d\ln S_{1/2}=\left[K_{1}\left(G_{11}-G_{21}\right)+K_{2}\left(G_{22}-G_{12}\right)\right]d\rho_{1}$$

where

24 $$K_{i}=\frac{\left\langle n_{i}\right\rangle }{v\rho_{i}}$$

is the interface/gas partition coefficient of the sorbate species *i*. Thus, simply by plotting ln *S*_1/2_ against ρ_1_, we can gain insights into net self-interaction, namely, the preferential self-interactions (gas 1-probe 1 and gas 2-probe 2) over mutual (gas 1-probe 2 and gas 2-probe1) (see Figure 4a). This net self-interaction, using eqs 10–12, 24, and 22, can be interpreted as

25 $$\begin{array}{l} d\;\ln S_{1/2}=d\;\ln\frac{\left\langle n_{1}\right\rangle }{\rho_{1}}-d\;\ln\frac{\left\langle n_{2}\right\rangle }{\rho_{2}}\\ d\;\ln\frac{\left\langle n_{1}\right\rangle }{\rho_{1}}=\left(K_{1}G_{11}-K_{2}G_{12}\right)d\rho_{1}\\ d\;\ln\frac{\left\langle n_{2}\right\rangle }{\rho_{2}}=\left(K_{1}G_{21}-K_{2}G_{22}\right)d\rho_{1} \end{array}$$

which signifies a competition between (i) preferential association of gas 1 with probe 1 and (ii) preferential association of gas 1 with probe 2 (see Figure 4b). These preferential interactions that competitively determine ln *S*_1/2_ can be quantified simply by plotting ln­(⟨*n*_*i*_⟩/ρ_*i*_) against ρ_1_ (see Figure 4b).

**Figure 4:**
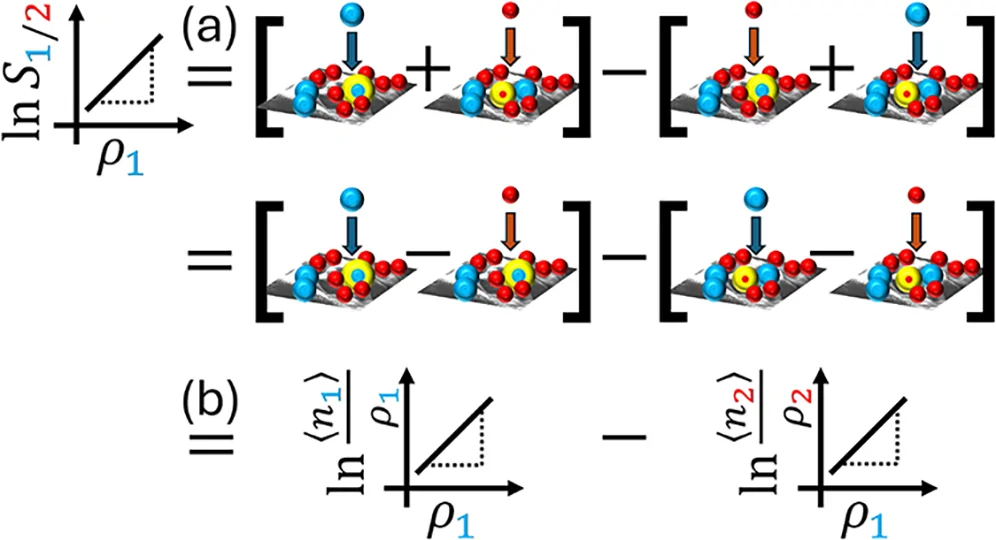
A schematic diagram for a model-free interpretation of selective sorption directly from experimental data for species 1 (blue) and 2 (red), as well as their probe molecules indicated by yellow. (a) By plotting ln *S*_1/2_ against ρ_1_, its gradient yields net self-interaction via eq 23. (b) The net self-interaction in (a) can be divided via eq 25 into the competitive contributions from (i) preferential association of gas 1 with probe 1 and (ii) preferential association of gas 1 with probe 2.

#### The ABC Isotherm

The ABC isotherm will also be simplified along a constant ρ. This can be appreciated by substituting eq 22 into the isotherm-generating relationship (eq 14), which yields

26 $$\begin{array}{l} d\frac{\rho_{1}}{\left\langle n_{1}\right\rangle }=\rho \;\left(\frac{G_{11}}{v}-S_{2/1}\frac{G_{12}}{v}\right)\;dy_{2}\\ d\frac{\rho_{2}}{\left\langle n_{2}\right\rangle }=\rho \;\left(\frac{G_{22}}{v}-S_{1/2}\frac{G_{21}}{v}\right)\;dy_{1} \end{array}$$

Likewise, the characteristic equation (eq 16) can be simplified under eq 22 to (see Supporting Information: D for derivation):

27 $$\begin{array}{l} \frac{G_{11}}{v}-S_{2/1}\frac{G_{12}}{v}=\Delta B_{1}^{\left(\rho ,0\right)}-\rho \Delta C_{1}^{\left(\rho ,0\right)}y_{2}\\ \frac{G_{22}}{v}-S_{1/2}\frac{G_{21}}{v}=\Delta B_{2}^{\left(0,\rho \right)}-\rho \Delta C_{2}^{\left(0,\rho \right)}y_{1} \end{array}$$

where

28 $$\begin{array}{l} \Delta B_{1}^{\left(\rho ,0\right)}\equiv B_{11}^{\left(\rho ,0\right)}-B_{12}^{\left(\rho ,0\right)}\\ \Delta B_{2}^{\left(0,\rho \right)}\equiv B_{22}^{\left(0,\rho \right)}-B_{21}^{\left(0,\rho \right)}\\ \Delta C_{1}^{\left(\rho ,0\right)}\equiv C_{111}^{\left(\rho ,0\right)}-2C_{112}^{\left(\rho ,0\right)}+C_{122}^{\left(\rho ,0\right)}\\ \Delta C_{2}^{\left(0,\rho \right)}\equiv C_{222}^{\left(0,\rho \right)}-2C_{221}^{\left(0,\rho \right)}+C_{211}^{\left(0,\rho \right)} \end{array}$$

will be interpreted in the next paragraph. Integrating eq 27 (see Supporting Information: D) yields

29 $$\begin{array}{l} \left\langle n_{1}\right\rangle =\frac{\rho y_{1}}{A_{1}^{\left(\rho ,0\right)}+\Delta B_{1}^{\left(\rho ,0\right)}\rho y_{2}-\frac{1}{2}\Delta C_{1}^{\left(\rho ,0\right)}\rho^{2}y_{2}^{2}}\\ \left\langle n_{2}\right\rangle =\frac{\rho y_{2}}{A_{2}^{\left(0,\rho \right)}+\Delta B_{2}^{\left(0,\rho \right)}\rho y_{1}-\frac{1}{2}\Delta C_{2}^{\left(0,\rho \right)}\rho^{2}y_{1}^{2}} \end{array}$$

which is the binary ABC isotherm along the constant total pressure.

#### Interpreting Isotherm Parameters

Here, we clarify the interpretations of the combined isotherm parameters (*A*_*i*_, Δ*B*_*i*_, and Δ*C*_*i*_) that appear in eq 29 along a constant ρ. First, 1/*A*_1_^(ρ,0)^ and 1/*A*_2_^(0,ρ)^, in addition to their rigorous origin as the surface-sorbate KBIs (see Supporting Information: D), can be interpreted (at low gas pressures) simply as the interface/gas partition coefficient, via

30 $$\frac{1}{A_{1}^{\left(\rho ,0\right)}}=vK_{1}^{\left(\rho ,0\right)},\frac{1}{A_{2}^{\left(0,\rho \right)}}=vK_{2}^{\left(0,\rho \right)}$$

Note that gas consists purely of species 1 at (ρ, 0) and purely of species 2 at (0,ρ), hence *A*_1_^(ρ, 0)^ and *A*_2_^(0, ρ)^ can be calculated from the parameters of the pure gas isotherms, via

$$\begin{array}{l} A_{1}^{\left(\rho ,0\right)}=A_{1}^{\left(0,0\right)}-B_{11}^{\left(0,0\right)}\rho -\frac{1}{2}C_{111}^{\left(0,0\right)}\rho^{2}\\ A_{2}^{\left(0,\rho \right)}=A_{2}^{\left(0,0\right)}-B_{22}^{\left(0,0\right)}\rho -\frac{1}{2}C_{222}^{\left(0,0\right)}\rho^{2} \end{array}$$ 31

Second, a simple interpretation for Δ*B*_1_^(ρ,0)^ and Δ*B*_2_^(0,ρ)^ follows eq 27

32 $$\begin{array}{l} \Delta B_{1}^{\left(\rho ,0\right)}={\left(\frac{G_{11}}{v}-S_{2/1}\frac{G_{12}}{v}\right)}^{\left(\rho ,0\right)}\\ \Delta B_{2}^{\left(0,\rho \right)}={\left(\frac{G_{22}}{v}-S_{1/2}\frac{G_{21}}{v}\right)}^{\left(0,\rho \right)} \end{array}$$

which signifies the preferential self-interaction over mutual. (See Supporting Information: D for the interpretation of Δ*B*/*A*.) Third, Δ*C*_1_^(ρ,0)^ and Δ*C*_2_^(0,ρ)^ in eq 29 can be interpreted (through tedious yet straightforward algebra in Supporting Information: D) via the conditional χ parameter, as

33 $$\begin{array}{l} \Delta C_{1}^{\left(\rho ,0\right)}={\left(\frac{\chi_{1}}{A_{1}}-\frac{{\left(\Delta B_{1}\right)}^{2}}{A_{1}}\right)}^{\left(\rho ,0\right)}\\ \Delta C_{2}^{\left(0,\rho \right)}={\left(\frac{\chi_{2}}{A_{2}}-\frac{{\left(\Delta B_{2}\right)}^{2}}{A_{2}}\right)}^{\left(0,\rho \right)} \end{array}$$

where

34 $$\chi_{i}\equiv \frac{\langle n_{i}\rangle_{i}N_{i,\mathrm{ii}}-\left\langle n_{i}\right\rangle N_{\mathrm{ii}}}{\langle n_{i}\rangle^{2}}-2S_{j/i}\frac{\langle n_{i}\rangle_{i}N_{i,\mathrm{ij}}-\left\langle n_{i}\right\rangle N_{\mathrm{ij}}}{\left\langle n_{i}\right\rangle \left\langle n_{j}\right\rangle }+S_{j/i}^{2}\frac{\langle n_{j}\rangle_{i}N_{i,\mathrm{jj}}-\left\langle n_{j}\right\rangle N_{\mathrm{jj}}}{\langle n_{j}\rangle^{2}}$$

is the net self-interaction (self-minus mutual) in the presence of a probe sorbate of species *i* (≠ *j*). Thus, Δ*C*_*i*_ signifies the enhancement of self-interaction around the probe sorbate *i* located at the interface.

#### Relationship to the Extended Langmuir Model

The binary ABC isotherm calls for a reconsideration of how sorbate interactions should be captured by an isotherm. This can be made clear by rewriting the binary ABC isotherm (along a constant pressure, eq 29) in the form mathematically analogous to the Extended Langmuir model, which has the following mathematical form:

35 $$\begin{array}{l} \left\langle n_{1}\right\rangle =\frac{n_{1}^{\left(L\right)}K_{11}^{\left(L\right)}\rho_{1}}{1+K_{11}^{\left(L\right)}\rho_{1}+K_{12}^{\left(L\right)}\rho_{2}}\\ \left\langle n_{2}\right\rangle =\frac{n_{2}^{\left(L\right)}K_{22}^{\left(L\right)}\rho_{2}}{1+K_{21}^{\left(L\right)}\rho_{1}+K_{22}^{\left(L\right)}\rho_{2}} \end{array}$$

where *n*_1_^(*L*)^ and *n*_2_^(*L*)^ are the “saturation capacities” of species 1 and 2, and *K*_*ij*_^(*L*)^ are the “Langmuir constants”, respectively. The remaining task is to rewrite the binary AB isotherm (along constant ρ; eq 29 with Δ*C*_*i*_ = 0) in the form of eq 35 (see Supporting Information: E), which yields

36 $$\begin{array}{l} \left\langle n_{1}\right\rangle =\frac{\rho_{1}}{A_{1}^{\left(0,0\right)}-B_{11}^{\left(\rho ,0\right)}\rho_{1}-B_{12}^{\left(\rho ,0\right)}\rho_{2}}\\ \left\langle n_{2}\right\rangle =\frac{\rho_{2}}{A_{2}^{\left(0,0\right)}-B_{21}^{\left(0,\rho \right)}\rho_{1}-B_{22}^{\left(0,\rho \right)}\rho_{2}} \end{array}$$

Comparing eq 36 with the Extended Langmuir (eq 35), we obtain the following correspondence (see Supporting Information: E):

37 $$\begin{array}{l} n_{1}^{\left(L\right)}=-\frac{1}{B_{11}^{\left(0,0\right)}},n_{2}^{\left(L\right)}=-\frac{1}{B_{22}^{\left(0,0\right)}}\\ K_{11}^{\left(L\right)}=-\frac{B_{11}^{\left(0,0\right)}}{A_{1}^{\left(0,0\right)}},K_{12}^{\left(L\right)}=\frac{\Delta B_{1}^{\left(\rho ,0\right)}-B_{11}^{\left(0,0\right)}}{A_{1}^{\left(0,0\right)}}\\ K_{22}^{\left(L\right)}=-\frac{B_{22}^{\left(0,0\right)}}{A_{2}^{\left(0,0\right)}},K_{21}^{\left(L\right)}=\frac{\Delta B_{2}^{\left(0,\rho \right)}-B_{22}^{\left(0,0\right)}}{A_{2}^{\left(0,0\right)}} \end{array}$$

Besides the truncation of the binary characteristic equations (eq 16), the binary AB isotherm is assumption-free, applicable to any pair of sorbates in arbitrary interfacial geometry or porosity. The Extended Langmuir model, in contrast, is a site-specific binding model for statistically independent binding sites on a homogeneous surface. Thus, the Extended Langmuir isotherm is a special, restricted case of the binary AB isotherm. Using this correspondence, we will identify what the Extended Langmuir model has failed to capture and how the ABC isotherm can rectify it in Results and Discussion.

Thus, we have successfully captured (i) surface-sorbate interactions via *A*_*i*_ and (ii) preferential self-association of sorbates via Δ*B*_*i*_ and Δ*C*_*i*_. We have replaced the local sorbate concentrations of the NRTL model with the sorbate number correlations using fluctuation theory (Figure 3), which does not need any model assumptions, such as lattice or cell models, interfacial geometry, or site-specific adsorption.

## Results and Discussion

### Model-Free Approach to Selective Sorption (Objective I)

The fluctuation theory enables a model-free mechanistic interpretation of selective sorption using experimental data alone. To demonstrate this, we used the published data on the adsorption of binary gas mixtures on activated carbon measured along a constant total gas pressure.^33^ Following our theory (eq 23), plotting the logarithm of the selectivity coefficient ln *S*_1/2_ against ρ_1_ yields the net-self-interaction as its gradient (Figure 5). (Note that the stronger adsorbed species of a pair is denoted as species 2 and the weaker one as 1.) For the pairs with weaker selectivity (e.g., ethylene-ethane), the gradient is near-zero. For the pairs with stronger selectivity (e.g., methane–ethane), the negative gradient, especially when the stronger-adsorbing species 2 is dilute, indicates preferential mutual interaction (i.e., 1–2 interactions are stronger than 1–1 and 2–2). The origin of this preferential mutual interaction can be identified by dissecting ln *S*_1/2_ into the sorption contributions of species 1 and 2 (via eq 25; Figure 6). For the pairs with stronger selectivity, the positive gradients of ln (⟨*n*_2_⟩/ρ_2_) (indicating a stronger 2–1 interaction over 2–2) override the smaller positive gradients of ln (⟨*n*_1_⟩/ρ_1_) (signifying a stronger 1–1 interaction over 1–2). Thus, the stronger adsorbate (species 2) associates strongly with species 1 and has a weaker tendency for self-association. This model-free insight, which derives directly from the fundamentals of fluctuation theory, will be clarified in the next subsection using the binary isotherm equations.

**Figure 5:**
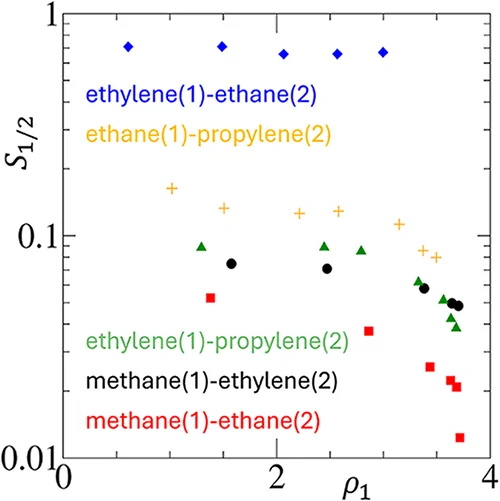
Model-free interpretation, via eq 23, of selectivity in the adsorption of binary gas mixtures (at 323 K at the total pressure 10 kPa) on activated carbon measured by Costa et al.^33^ for ethylene(1)-ethane(2) (blue diamonds), ethane(1)-propylene(2) (orange crosses), ethylene(1)-propylene(2) (green triangles), methane(1)-ethylene(2) (black squares), and methane(1)-ethane(2) (red circles). According to eq 23, the negative gradient signifies the preference for 1–2 association (see Figure 4a).

**Figure 6:**
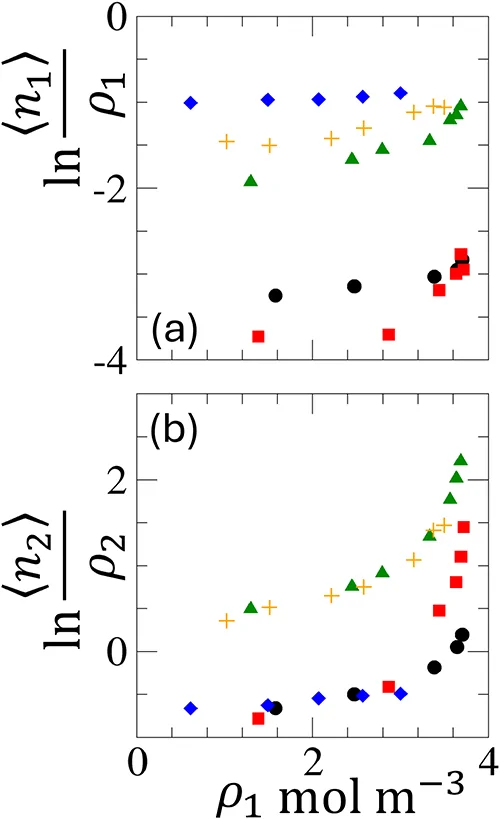
Model-free interpretation of selectivity, divided, via eq 25, into the two competing interactions (see Figure 4b): the preferential interaction of gas 1 (over gas 2) with probe 1 from the positive gradient of (a) and the preferential interaction of gas 1 (over gas 2) with probe 2 as shown in the positive gradient of (b). A steeper gradient in (b) shows its dominant contribution to Figure 5. The symbols and colors were defined in the caption to Figure 5.

### Modeling Selective Sorption via the ABC Isotherm (Objective II)

#### Pure Gas Isotherms

Our goal is to model selective sorption via the ABC isotherm. In this context, studying the sorption isotherms of pure gases serves the following two purposes. First, the ABC isotherm (eq 18) can fit the sorption isotherms of pure gases on activated carbon (Figure 7) only with *A*_1_^(0,0)^ and *B*_11_^(0,0)^ (i.e., under *C*_111_^(0.0)^ = 0), thereby providing the initial testing of the ABC isotherm (eq 18). The fitting parameters (Table 2) reveal attractive surface-sorbate interaction (*A*_1_^(0,0)^ > 0) and repulsive disorbate (sorbate–sorbate) interactions at the interface (*B*_11_^(0,0)^ < 0). We emphasize that the AB isotherm incorporates sorbate sizes naturally via negative *B*_11_^(0,0)^, without introducing model assumptions. Second, two of the parameters (*A*_1_^(ρ,0)^ and *A*_2_^(0,ρ)^) for the binary ABC isotherm (eq 29) along a constant total pressure (summarized in Table 3) can be evaluated (using eq 31) from *A*_1_^(0,0)^ and *B*_11_^(0,0)^. Thus, the AB isotherm was demonstrated to model pure gas sorption and provided two of the parameters required for modeling selective sorption.

**Figure 7:**
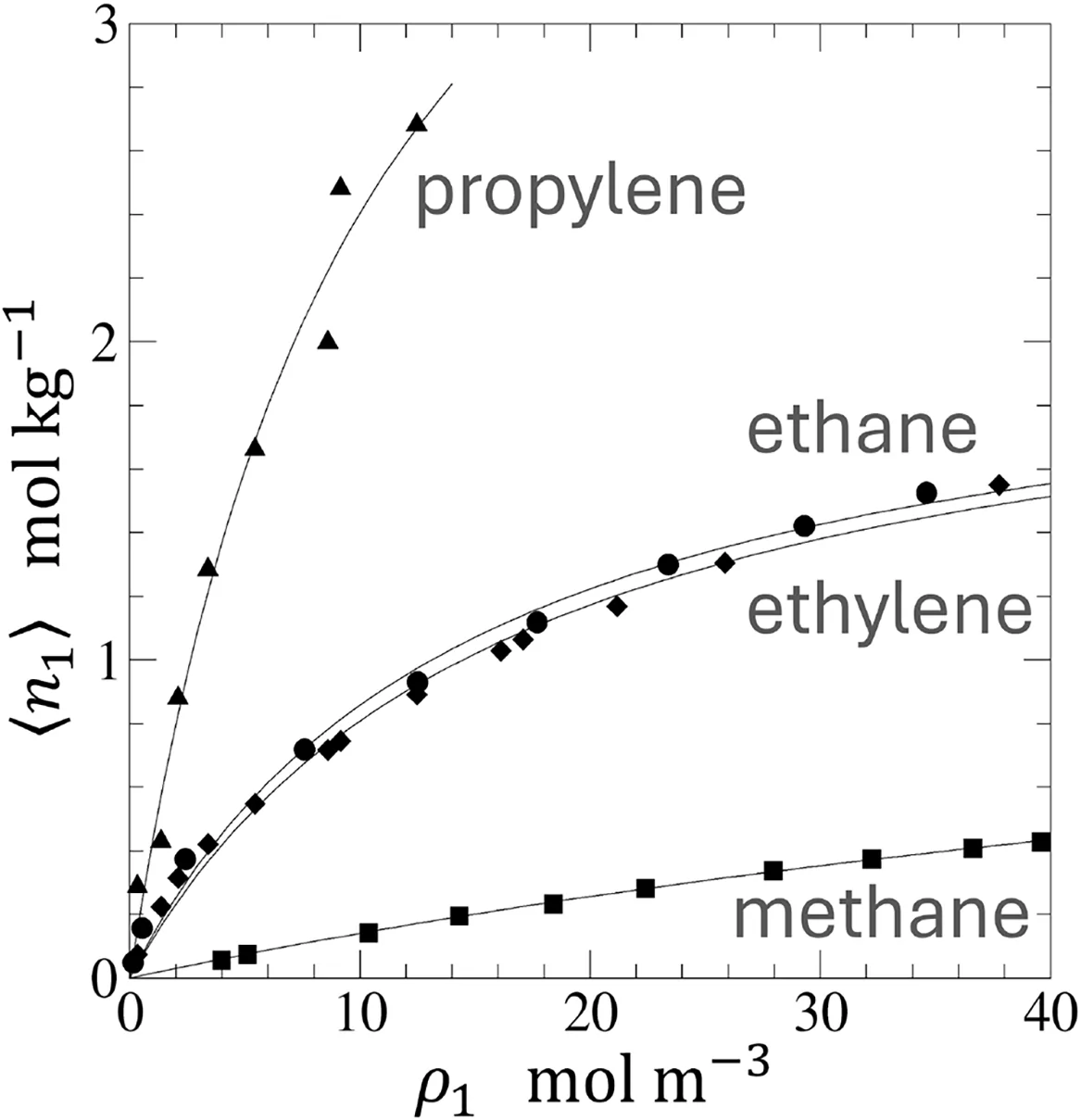
Adsorption isotherms of pure gases on activated carbon measured by Costa et al.^33^ at 323 K for methane (squares), ethane (circles), ethylene (diamonds), and propylene (triangles). The solid lines represent fittings by the AB isotherm (eq 18; with the parameters summarized in Table 2). The horizontal axis is ρ_1_ = *P*_1_/*RT* (in mol m^–3^), and the vertical axis is the amount of sorption (in mol kg^–1^).

**Table 2:** AB Isotherm Parameters for Pure Gas Adsorption on Activated Carbon with *C*_111_^(0,0)^ = 0

sorbate (1)	*A*_1_^(0,0)^/kg m^–3^	*B*_11_^(0,0)^/kg mol^–1^
methane	64.2	–0.693
ethane	6.96	–0.470
ethylene	7.69	–0.468
propylene	2.10	–0.206

**Table 3:** Parameters for the Binary ABC Isotherm (eq 29)­^a^

sorbate (1)	sorbate (2)	*A*_1_^(ρ,0)^ ^b^	*A*_2_^(0,ρ)^ ^b^	Δ*B*_1_^(ρ,0)^ ^c^	Δ*B*_2_^(0,ρ)^ ^c^	Δ*C*_1_^(ρ,0)^ ^d^	Δ*C*_2_^(0,ρ)^ ^d^
ethylene	ethane	9.44	8.71	–0.392	–2.04	–0.545	–0.862
ethane	propylene	8.71	2.87	6.08	–0.167	2.44	0.230
ethylene	propylene	10.4^e^	2.87	12.2	–0.209	6.12	0.224
methane	ethylene	66.8	9.44	27.8	–1.31	14.7	0.0636
methane	ethane	66.8	8.71	85.8	0.998	27.7	1.62

a At ρ = 3.72 mol m^–3^ (total pressure = 10 kPa).

b Units are in kg m^–3^. Calculated from pure gas isotherms via eq 31 with *C*_111_^(0,0)^ = 0.

c Units are kg mol^–1^.

d Units are kg m^3^ mol^–2^.

e Only for this pair, *A*_1_^(ρ,0)^ had to be obtained as a fitting parameter, which deviated slightly from 9.44 as evaluated from pure gas isotherm.

#### ABC Isotherms for Mixtures Along Constant Pressure

The binary ABC isotherm (eq 29) can model the sorption isotherms of both sorbates along a constant total pressure, as has been demonstrated in Figure 8. Since *A*_1_^(ρ,0)^ and *A*_2_^(0,ρ)^ have already been determined from pure isotherms (Figure 7), the remaining parameters to be determined are (i) Δ*B*_1_^(ρ,0)^, Δ*B*_2_^(0,ρ)^, Δ*C*_1_^(ρ,0)^, and Δ*C*_2_^(0,ρ)^ for the ABC isotherm (eq 29; Table 3) or (ii) only Δ*B*_1_^(ρ,0)^ and Δ*B*_2_^(0,ρ)^ for the AB isotherm (eq 29 with Δ*C*_1_^(ρ,0)^ = Δ*C*_2_^(0,ρ)^ = 0; see Table 4). From the isotherm fitting, how the sorbate mole fraction *x*_1_ at the interface,

38 $$x_{1}\equiv \frac{\left\langle n_{1}\right\rangle }{\left\langle n_{1}\right\rangle +\left\langle n_{2}\right\rangle }$$

changes with the gas composition *y*_1_,

39 $$y_{1}\equiv \frac{\rho_{1}}{\rho_{1}+\rho_{2}}$$

can be evaluated (Figure 9). In these figures, excellent agreement with experiments has been observed for the ABC isotherms despite the relatively small number of parameters, without any complications arising from implicit function relationships (see Supporting Information: F). Albeit less accurately than the ABC, it is striking that even the AB isotherm was able to capture selective sorption. Thus, we have demonstrated that the ABC isotherm can accurately model binary sorption isotherms along a constant total pressure.

**Figure 8:**
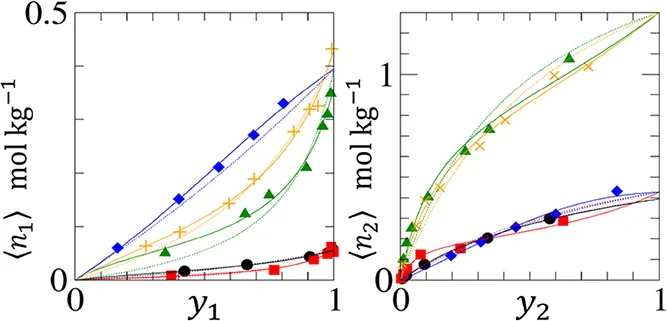
Adsorption isotherms on activated carbon of the same pairs of gases as Figure 5. The panels (a) and (b) show the adsorption isotherms of species 1 and 2, plotted against the corresponding mole fractions in the gas phase, *y*_*i*_, respectively. The fittings via ABC (solid lines, eq 29) and AB (dotted lines, eq 29 with Δ*C*_1_^(ρ,0)^ = Δ*C*_2_^(0,ρ)^ = 0) isotherms are shown in the same colors as Figure 5. The fitting parameters for the ABC and AB isotherms are summarized in Tables 3 and 4, respectively.

**Figure 9:**
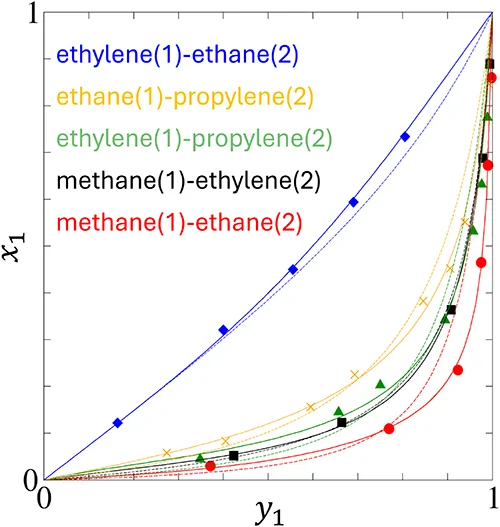
Interfacial mole fraction *x*_1_ (eq 38) against gas mole fraction *y*_1_ (eq 39) evaluated from experimental data (same symbols as Figure 5) with the calculations from the ABC (solid lines) and AB (dashed lines).

**Table 4:** Parameters for the Binary AB Isotherm (eq 29 with Δ*C*_1_^(*ρ*,0)^ = Δ*C*_2_^(0,*ρ*)^ = 0)^a^

sorbate (1)	sorbate (2)	*A*_1_^(ρ,0)^ ^b^	*A*_2_^(0,ρ)^ ^b^	Δ*B*_1_^(ρ,0)^ ^c^	Δ*B*_2_^(0,ρ)^ ^c^
ethylene	ethane	9.44	8.71	0.653	–1.09
ethane	propylene	8.71	2.87	4.66	–0.469
ethylene	propylene	9.44	2.87	13.0	–0.520
methane	ethylene	66.8	9.44	17.3	–1.40
methane	ethane	66.8	8.71	77.1	–1.20

a At ρ = 3.72 mol m^–3^ (total pressure = 10 kPa).

b Units are in kg m^–3^. Calculated from pure gas isotherms via eq 31 with *C*_111_^(0,0)^ = 0.

c Units are kg mol^–1^.

#### Algorithmic Clarity of the Binary ABC Isotherm

The binary ABC isotherm is a closed-form, explicit function, which requires only standard nonlinear regression to determine all its parameters with a direct link to the distribution of sorbate molecules. In contrast to the activity models (such as IAST^27–29^ or RAST^16,30–33^ ) and to the Generalized Langmuir model,^9,15,16^ the binary ABC isotherm does not require the point-by-point nested iterative procedure that arises from solving a system of implicit, mutually coupled relationships. Thus, the binary ABC isotherm achieves accurate modeling of sorption selectivity (as demonstrated above) without resorting to the nested iterative procedures required by IAST,^27–29^ RAST,^16,30–33^ or Generalized Langmuir.^9,15,16^

### Capturing Sorbate Attractions and Repulsions (Objective III)

#### Mechanistic Insights from Isotherm Parameters

Not only can the ABC isotherm fit the sorption data but it also clarifies the selective sorption mechanism from its parameters. We will clarify what drives a deviation from *x*_1_ = *y*_1_ in Figure 9. There is a clear trend linking the selectivity (Figure 9) and the AB isotherm parameters (Table 4). The stronger the selectivity (i.e., the deviation from the nonselectivity *x*_1_ = *y*_1_ line), the stronger the disparity becomes both in sorbate-surface interaction (1/*A*_1_^(ρ,0)^ versus 1/*A*_2_^(0,ρ)^) and in sorbate–sorbate interactions (Δ*B*_1_^(ρ,0)^ versus Δ*B*_2_^(0,ρ)^) from the weakest ethylene-ethane pair to the strongest methane–ethane. While propylene exhibits the strongest surface interaction (smallest *A*), interaction disparity is minor for both the ethane-propylene and ethylene-propylene pairs. In contrast, ethylene and ethane have modest surface interaction (intermediate *A*) yet become highly selective when paired with methane (which interacts the weakest with the surface). The curvature of the *y*_1_-*x*_1_ plot is driven by a large disparity in Δ*B*. Note that methane is the smallest sorbate in this data set, whose size (i.e., small excluded volume) may be the reason for its strongest preferential self-association (i.e., large positive Δ*B*_1_^(ρ,0)^). Understanding the shape of the selectivity plot (Figure 9) will be made clearer in the next subsection when we clarify the origin of the individual isotherm shapes via the AB parameters.

#### Preferential Sorbate Self-Association Identifiable from the Isotherm Shapes

Under constant total gas concentration (ρ = ρ_1_ + ρ_2_ = const), the isotherms for species 1 and 2 depend only on the gas composition (*y*_1_ defined by eq 39 and *y*_2_ = 1 – *y*_1_). These isotherms, namely, ⟨*n*_1_⟩(*y*_1_) and ⟨*n*_2_⟩(*y*_2_) shown in Figure 8, are similar in shape to IUPAC Types III and I for unary isotherms, respectively. (See Supporting Information: B for how the ABC isotherm provides a mechanistic foundation for the understanding and classification of unary isotherms.) We have recently shown that sorbate–sorbate attraction versus repulsion distinguishes single-sorbate Types III and I.^41,54^ Does a similar logic hold for our binary isotherms in Figure 8? To answer this question, we rewrite our binary AB isotherm in a form mathematically analogous to the single-component counterpart, via

40 $$\left\langle n_{i}\right\rangle =\frac{\rho_{i}}{\tilde{A_{i}}-\tilde{B_{i}}\rho_{i}}$$

where

41 $$\begin{array}{l} \tilde{A_{1}}=A_{1}^{\left(\rho ,0\right)}+\rho \Delta B_{1}^{\left(\rho ,0\right)},\tilde{A_{2}}=A_{2}^{\left(0,\rho \right)}+\rho \Delta B_{2}^{\left(0,\rho \right)}\\ \tilde{B_{1}}=\Delta B_{1}^{\left(\rho ,0\right)},\tilde{B_{2}}=\Delta B_{2}^{\left(0,\rho \right)} \end{array}$$

with a generalization to the ABC isotherm presented in Supporting Information: E. We emphasize that $\tilde{B_{1}}$ and $\tilde{B_{2}}$ , calculated using eq 41, can be positive or negative, representing net-self-association and net-self-dissociation, respectively. Equation 41 rationalizes why ⟨*n*_1_⟩(ρ_1_) is Type III-like while ⟨*n*_2_⟩(ρ_2_) is Type I-like. For ⟨*n*_1_⟩(ρ_1_), the apparent surface-sorbate 1 interaction, $1/\tilde{A_{1}}$ , is small, because not only is sorbate-surface interaction already weak (judging from 1/*A*_1_^(ρ,0)^ < 1/*A*_2_^(0,ρ)^) but also sorbate 2–1 interaction is weaker than 1–1 (i.e., Δ*B*_1_^(ρ,0)^ > 0), making surface-sorbate 1 interaction effectively weaker because the surface is populated predominantly by species 2 at *y*_1_ ≃ 0 (Table 5). However, the same Δ*B*_1_ > 0 causes the cooperative increase of ⟨*n*_1_⟩ with ρ_1_. Thus, preferential self-association of sorbate 1 drives the Type III-like behavior of ⟨*n*_1_⟩(ρ_1_) (Table 5). In contrast, the Type I-like behavior of ⟨*n*_2_⟩(ρ_2_) comes from a strong sorbate 2-surface interaction (1/*A*_2_^(0,ρ)^), which is enhanced further by a preferential sorbate 2–1 association (because sorbates at *y*_2_ ≃0 are predominantly species 1). In addition, the preferential 2–1 association, i.e., $\tilde{B_{2}}<0$ , is responsible for the Type I-like saturation of ⟨*n*_2_⟩(ρ_2_) (Table 5). Thus, the isotherm shapes are determined by preferential self-association versus dissociation of sorbates, as a natural generalization of how sorbate attraction versus repulsion underlie the IUPAC isotherm type difference for pure sorbates.^41,53,54,61,64,65,70,74,75^

**Table 5:** Understanding Isotherm Shape via eq 41

sorbate (1)	sorbate (2)	$1/\tilde{A_{1}}$ ^a^	$1/\tilde{A_{2}}$ ^a^	$\tilde{B_{1}}$ ^a^	$\tilde{B_{2}}$ ^a^
ethylene	ethane	0.0842	0.215	0.653	–1.09
ethane	propylene	0.0384	0.890	4.66	–0.469
ethylene	propylene	0.0173	1.07	13.0	–0.520
methane	ethylene	0.00762	0.237	17.3	–1.40
methane	ethane	0.00283	0.236	77.1	–1.20

a Calculated from Table 4 using eq 41.

#### Langmuirian Models Cannot Capture Sorbate Attraction

We have demonstrated that the key to understand selective sorption is to incorporate both the attraction and the repulsion between sorbates. The AB isotherm could successfully account for both attraction and repulsion while retaining the mathematical simplicity analogous to the Extended Langmuir model (eq 37). This raises a question: what could the AB isotherm capture while the Extended Langmuir could not? To answer this question, since the extended Langmuir isotherm is a special, restricted case of the binary AB isotherm, let us map the AB parameters to those of the Extended Langmuir, via eq 37. Strikingly, *K*_21_^(*L*)^ is negative (see Table 6), in contradiction to its Langmuirian definition as an association constant. This means that the experimental binary isotherms cannot be modeled by the Extended Langmuir isotherm. However, it is not surprising for our theory to have negative *K*_21_^(*L*)^, because it is a simple consequence of sorbate 1-sorbate 2 attraction at *y*_1_ = 1 (i.e., *B*_21_^(0,ρ)^ > 0, since *A*_1_^(0,0)^ and *A*_2_^(0,0)^ are both positive). This is reminiscent of the historic difficulties in modeling Type III single-sorbate isotherms through the Langmuir, BET, and GAB models.^54^ These classical models assumed the sorbate binding sites to be distributed uniformly on the surface, hence inadvertently focused on sorbate–sorbate repulsion; indeed, two sorbates are separated when they are bound at two separate sites.^41^ However, the attempts have long been focused on how to incorporate “lateral” interactions between sorbate molecules, thereby perpetuating the asymmetrical treatment of sorbate–sorbate attraction and repulsion.^41,54^ Thus, the historic difficulties of the multicomponent Langmuir models in capturing selective sorption originate from their unequal treatment of sorbate–sorbate attraction and repulsion.

**Table 6:** Breakdown of the Extended Langmuir Model^a^

sorbate (1)	sorbate (2)	*n*_1_^(*L*)^	*n*_2_^(*L*)^	*K*_11_^(*L*)^	*K*_12_^(*L*)^	*K*_21_^(*L*)^	*K*_22_^(*L*)^
ethylene	ethane	2.14	2.13	0.0609	0.146	–0.0891	0.0675
ethane	propylene	2.13	4.85	0.0675	0.737	–0.125	0.0981
ethylene	propylene	2.14	4.85	0.0609	1.75	–0.148	0.0981
methane	ethylene	1.44	2.14	0.0108	0.280	–0.121	0.0609
methane	ethane	1.44	2.13	0.0108	1.21	–0.105	0.0675

a Calculated from Tables 2 and 4 using eq 37.

## Conclusions

Our goal was to elucidate the mechanism of gas separation via sorption through quantifying underlying interactions between the surface and sorbates, and between sorbate species sorbed at the interface. We have shown how these interactions can be quantified from experimental sorption isotherms alone, guided directly by the principle of statistical thermodynamic fluctuation theory, without introducing any models or assumptions. This was achieved by generalizing our theory for pure sorbates^41,53,54,61,64,65^ to sorbate mixtures, thereby establishing (1) the fundamental, model-free relationships for competitive sorption, (2) the ABC sorption isotherm for binary sorbate mixtures, and (3) adaptation of (1) and (2) for sorption selectivity along a constant total pressure.

With these three novel tools (1–3), we have demonstrated that (I) the preferential self-interactions of gases at the surface, that determine sorption selectivity, can be elucidated from experimental data alone without any model assumptions; (II) the ABC isotherm successfully models competitive sorption of binary mixtures and quantifies the key interactions (i.e., surface-sorbate and sorbate–sorbate interactions) for sorption selectivity as its parameters; and (III) the capacity of handling sorbate–sorbate attraction and repulsion at the interface is crucial for elucidating selective sorption.

Our novel approach has achieved what previous models tried to achieve, while overcoming their complications. The fluctuation theory could capture local sorbate inhomogeneities (e.g., preferential self-interaction) without requiring model constructs (such as local concentrations or phantom molecules). The excess numbers and KBIs, derived directly from number correlations, serve as the universal language for interactions at interfaces and in liquid solutions.^76^

Our ABC isotherm is founded on KBIs, whose parameters incorporate local inhomogeneity in sorbate distributions. This is in contrast to the classical isotherm models (e.g., Langmuir, BET, and GAB) whose parameters represent site-specific binding phenomena (e.g., binding constants and the number of sites), which required consistency with the activity models for adsorbates at the interface. Such consistency criteria, with complications from implicit function relationships, have been made redundant by our theory.

Like the preceding theory for pure sorbates,^41,53,54,61^ our theory for sorbate mixtures will cover any interfacial geometry, porosity, or molecular size and shape, valid for “localized” and “mobile” adsorptions^58^ alike, overcoming the long-standing restrictions of Langmuirian models^19,20^ to site-specific adsorption on uniform surfaces. In a forthcoming paper, we plan to generalize our theory on the temperature dependence of sorption^77^ to selective and competitive sorption isotherms.

## Supplementary Material



## Notes

Setting up the interface; unary ABC isotherm and its relationship to IUPAC classifications; sorption of gas mixtures; isotherms along a constant total pressure; understanding binary sorption isotherm shapes; goodness of fit values for the ABC and AB isotherms (PDF)
