# Supplementary material for: Gas Separation by Sorption: A Statistical Thermodynamic Fluctuation Theory
Source: Langmuir. 2026 Aug 31;42(36):26214–26. doi: 10.1021/acs.langmuir.6c02601 (PMC13585139; doi:10.1021/acs.langmuir.6c02601)
Supplement: Supplementary file 1 [file la6c02601_si_001.pdf]

Supporting Information

Gas Separation by Sorption: A Statistical Thermodynamic Fluctuation Theory

Seishi Shimizu*,† and Nobuyuki Matubayasi‡

†*York Structural Biology Laboratory, Department of Chemistry, University of York, Heslington, York YO10 5DD, United Kingdom*

‡*Division of Chemical Engineering, Graduate School of Engineering Science, Osaka University, Toyonaka, Osaka 560-8531, Japan*

E-mail: seishi.shimizu@york.ac.uk

Contents

A Setting Up the Interface **S2**

B The Unary ABC Isotherm and Its Relationship to IUPAC Classifications **S5**

C Sorption of Gas Mixtures **S10**

D Isotherms Along a Constant Total Pressure **S15**

E Understanding Binary Sorption Isotherm Shapes **S20**

F Goodness of Fit (R^2) Values for the ABC and AB Isotherms **S22**

Note: Reference numbers in this Supporting Information correspond to those in the main text.

A Setting Up the Interface

A.1 Surface Excess and Amount Adsorbed

Here, we generalize our recent work[61] to set up the solid/gas interface of arbitrary interfacial geometry, including pores and crevices. We consider a $\tau + 1$-component system consisting of sorbent (species e) and sorbate species k ($k = 1, \cdots, \tau$). Following Gibbs[67] and our previous papers,[64,66] an interface is defined as the difference between the system (denoted by $*$) and the two reference systems on the solid (denoted by I) and the gas sides (denoted by II). The interfacial free energy F is the difference in grand thermodynamic functions (Ω) between the system (without superscript) and the reference systems on the solid (s) and the bulk gas (b) sides,

$$\Omega = -PV + F, \qquad \Omega^s = -PV^s, \qquad \Omega^b = -PV^b \tag{A.1}$$

where V is the volume. Note that the system and the references have the same pressure P.[64,66] To introduce the surface, the conventional approach introduces the Gibbs dividing surface so as to satisfy

$$N_e = N_e^s + N_e^b \tag{A.2}$$

However, such a condition (A.2) can be introduced purely algebraically via the Legendre transform. To do so, we carry out the Legendre transform ($Y = \Omega + \mu_e N_e$) to introduce partially open ensembles closed only to e. Under phase equilibrium (equal μ_e in the three phases) and volume conservation $V = V^s + V^b$, eqs A.1 and A.2, in combination, become

$$F = Y - Y^s - Y^b \tag{A.3}$$

The differential form of eq A.3 is

$$dF = \sum_{k=1}^{\tau} \left(\langle N_k \rangle - \langle N_k^s \rangle - \langle N_k^b \rangle \right) d\mu_k \tag{A.4}$$

Conventionally, sorption is measured per unit mass of sorbent. Consequently,

$$\frac{1}{\langle N_e^s \rangle} dF = \sum_{k=1}^{\tau} \Gamma_k d\mu_k \tag{A.5}$$

where the surface excess is defined via

$$\Gamma_k = \frac{\langle N_k \rangle - \langle N_k^s \rangle - \langle N_k^b \rangle}{\langle N_e^s \rangle} \tag{A.6}$$

Equation A.5 is the multicomponent version of the generalized Gibbs isotherm. Since the interfacial effects are confined within the finite distance range from the interface,[64,65] eq A.6 can be written in a local form as

$$\Gamma_k = \frac{\langle n_k \rangle - \langle n_k^s \rangle - \langle n_k^b \rangle}{\langle N_e^s \rangle} \tag{A.7}$$

where $\langle n_k \rangle$ is the number of sorbate species k within the interfacial volume v, whereas $\langle n_k^s \rangle$ and $\langle n_k^b \rangle$ are the corresponding numbers within v of the reference systems. For the sorption isotherms that can be measured gravimetrically, $\langle n_k \rangle \gg \langle n_k^s \rangle, \langle n_k^b \rangle$ is satisfied (see Ref [53]). Under this condition, the surface excess reduces to the amount sorbed, i.e.,

$$\Gamma_k \simeq \frac{\langle n_k \rangle}{\langle N_e^s \rangle} \tag{A.8}$$

In the following, we will use $\langle n_k \rangle$ in place of $\frac{\langle n_k \rangle}{\langle N_e^s \rangle}$, i.e., $\langle n_k \rangle$ will be used to express the amount sorbed per unit mass of sorbent.

A.2 The Fluctuation Theory and the Thermodynamic Consistency

Conventional isotherm models, such as the Langmuir and BET, have not been derived from the Gibbs isotherm. Consequently, it was necessary to check whether these models were compatible with the multiple-component Gibbs isotherm (i.e., a special case of our A.5).[22] In contrast, our isotherms have a direct link with the Gibbs isotherm, via

$$\left(\frac{\partial}{\partial \mu_i}\frac{F}{\langle N_e^s\rangle}\right)_{\mu_{k\neq i}} = \Gamma_i \tag{A.9}$$

For the gravimetrically measurable isotherms (i.e., $\Gamma_i \simeq \langle n_i\rangle/\langle N_e^s\rangle$), the application of Maxwell's relationship on the generalized Gibbs isotherm (eq A.5) yields

$$\left(\frac{\partial\langle n_i\rangle}{\partial \mu_j}\right)_{\mu_{k\neq i,j}} = \left(\frac{\partial\langle n_j\rangle}{\partial \mu_i}\right)_{\mu_{k\neq i,j}} \tag{A.10}$$

Using the elementary statistical thermodynamic relationship,

$$k_BT\left(\frac{\partial\left\langle n_i\right\rangle}{\partial \mu_j}\right)_T = \langle \delta n_i \delta n_j\rangle \tag{A.11}$$

where $\delta n_i = n_i - \langle n_i\rangle$ is the deviation from the mean. Combining eqs A.10 and A.11, we obtain

$$\langle \delta n_i \delta n_j\rangle = \langle \delta n_j \delta n_i\rangle \tag{A.12}$$

which is satisfied automatically.

We emphasize that our isotherm equations are derived from the isotherm generating relationship based on eq A.11. The "thermodynamic consistency" of our isotherms is guaranteed by the symmetry relationship (eq A.12). The connection between eq A.11 and eq 5 of the

main text can be achieved by introducing the excess number, N_{ij}, defined via

$$N_{ij} = \frac{\langle \delta n_i \delta n_j \rangle - \delta_{ij} \langle n_i \rangle}{\langle n_i \rangle} \tag{A.13}$$

which is linked, via the logarithmic gradient of an isotherm, to the sorbate excess number, as shown in eq 5.

B The Unary ABC Isotherm and Its Relationship to IUPAC Classifications

This section aims to demonstrate how the unary ABC isotherm can be derived directly from the statistical thermodynamic fluctuation theory (Section B.1 - B.3), why its parameters are general, not restricted to pre-supposed sorption models (Section B.2), and how it provides a universal theoretical foundation for elucidating IUPAC Types I-III isotherms (Section B.4) including supercritical conditions (Section B.5). The unary isotherm can readily be extended to sorption from solution (Section B.6). The temperature dependence of sorption can be interpreted mechanistically via the fluctuation theory (Section B.7).

B.1 Isotherm-Generating Relationship

Here, we present a derivation of sorption isotherms of pure gases directly from the fluctuation theory. We take a different approach from our previous papers, aiming at the ease of generalization to multicomponent sorbates. Note that the saturation pressure (used to calculate sorbate activity) is no longer necessary, resulting in a direct relationship with the surface-sorbate KBI. Under constant temperature,

$$d \ln \langle n_1 \rangle = \left(\frac{\partial \ln \langle n_1 \rangle}{\partial \mu_1} \right)_T d\mu_1 \tag{B.1}$$

governs isotherm increase $d\ln\langle n_1\rangle$. With the help of the fluctuation relationship (eq 5), we obtain

$$d\ln\langle n_1\rangle = (N_{11}+1)\,d\ln\rho_1 \tag{B.2}$$

which, through straightforward algebra, converts to

$$d\ln\frac{\langle n_1\rangle}{\rho_1} = N_{11}d\ln\rho_1 \tag{B.3}$$

From eq B.3, we obtain

$$-\frac{d}{d\rho_1}\frac{\rho_1}{\langle n_1\rangle} = \frac{N_{11}}{\langle n_1\rangle} \tag{B.4}$$

which is the isotherm-generating relationship for the single-component ABC isotherm.

B.2 ABC Characteristic Equation

To derive an isotherm, we need to specify the functional form of $N_{11}/\langle n_1\rangle$. The simplest is the polynomial expansion in terms of ρ_1 around $\rho_1=\rho_1^r$, via

$$\frac{N_{11}}{\langle n_1\rangle} = B_{11}^r + C_{111}^r(\rho_1-\rho_1^r) \tag{B.5}$$

where B_{11}^r and C_{111}^r are the expansion coefficients defined at $\rho_1=\rho_1^r$. With the help of the sorbate-sorbate KBI at the interface, G_{11}, defined via eqs 15 and 20, B_{11}^r can be expressed as

$$B_{11}^r = \left(\frac{N_{11}}{\langle n_1\rangle}\right)_{\rho_1=\rho_1^r} = \left(\frac{G_{11}}{v}\right)_{\rho_1=\rho_1^r} \tag{B.6}$$

which signifies the sorbate-sorbate interaction at the interface. C_{111}^r is defined as

$$C_{111}^r = \left(\frac{\partial}{\partial\rho_1}\frac{N_{11}}{\langle n_1\rangle}\right)_{\rho=\rho_1^r} \tag{B.7}$$

which, with the help of eq 5, can be evaluated as

$$\begin{aligned} C_{111}^{r} &= \left(\frac{1}{\rho_1} \frac{\partial}{\partial \ln \rho_1} \frac{\langle n_1 \rangle_1 - \langle n_1 \rangle}{\langle n_1 \rangle} \right)_{\rho_1 = \rho_1^r} \\ &= \left\{ \frac{\langle n_1 \rangle}{\rho_1^r} \left[\frac{(\langle \delta n_1 \delta n_1 \rangle_1 - \langle n_1 \rangle_1) - (\langle \delta n_1 \delta n_1 \rangle - \langle n_1 \rangle)}{\langle n_1 \rangle^2} - \frac{(\langle n_1 \rangle_1 - \langle n_1 \rangle)^2}{\langle n_1 \rangle^2} \right] \right\}_{\boldsymbol{\rho} = \boldsymbol{\rho}^r} \end{aligned} \tag{B.8}$$

B.3 ABC Isotherm for Pure Sorbates

Integrating eqs B.4 and B.5 yields

$$\frac{\rho_1}{\langle n_1 \rangle} = A_1^{\rho^r} - B_{11}^{\rho^r} \left(\rho_1 - \rho_1^{\rho^r} \right) - \frac{C_{111}^{\rho^r}}{2} \left(\rho_1 - \rho_1^{\rho^r} \right)^2 \tag{B.9}$$

where $A_1^{\rho^r}$ is an integration constant whose interpretation will be clarified shortly. Equation B.9 can be transformed into the following form:

$$\langle n_1 \rangle = \frac{\rho_1}{A_1^{\rho^r} - B_{11}^{\rho^r} \left(\rho_1 - \rho_1^{\rho^r} \right) - \frac{C_{111}^{\rho^r}}{2} \left(\rho_1 - \rho_1^{\rho^r} \right)^2} \tag{B.10}$$

which is the ABC isotherm for pure gas sorbates, which uses the sorbate gas pressure $\rho_1 = P_1/RT$, instead of its activity a_1, as its variable. A special case of eq B.10 at $\rho^r = 0$,

$$\langle n_1 \rangle = \frac{\rho_1}{A_1^0 - B_{11}^0 \rho_1 - \frac{C_{111}^0}{2} \rho_1^2} \tag{B.11}$$

has been referred to as the "ABC isotherm", which contains the Langmuir, BET, and GAB isotherms as its special cases.

To interpret the parameter, A_1^r, in the ABC isotherm (eq B.10), we start by recognizing that $\langle n_1 \rangle$ came in as an approximation of Γ_1. Consequently,

$$\frac{1}{A_1^r} = \left(\frac{\Gamma_1}{\rho_1} \right)_{\rho_1^r} \simeq \left(\frac{\langle n_1 \rangle}{\rho_1} \right)_{\rho_1^r} \tag{B.12}$$

whose interpretation will be made clearer when the ideal gas equation of state can be applied to $\rho_1 = P_1/RT \simeq \langle n_1^b \rangle / v$, leading to

$$\frac{1}{A_1^r} \simeq \left(v \frac{\langle n_1 \rangle - \langle n_1^b \rangle}{\langle n_1^b \rangle} \right)_{\rho_1^r} = G_{s1}^r \tag{B.13}$$

which is the surface-sorbate KBI, G_{s2}^r, at $\rho = \rho^r$. Thus, adopting $\rho_1 = P_1/RT$ as the variable instead of sorbate activity a_1 leads to a clearer and more direct link between $1/A_1^r$ and the surface-sorbate KBI.

B.4 IUPAC Classification and the ABC Isotherm

In our recent paper,[54] we have demonstrated that the ABC isotherm can fit IUPAC Types I-III,[44] while our other isotherm, the cooperative isotherm, can fit IV-VI.[70,74,75] We emphasize that even modeling Types I-III posed difficulties for traditional models: (i) Type III cannot be captured by Langmuir, BET, or GAB models without choosing an unphysical parameter range (i.e., negative Langmuir or BET constants),[54] (ii) Since the Langmuir model is not a special case of the standard BET model used for vapor adsorption, the traditional models have had difficulties to fit experimental isotherms (e.g., micropores) that fall in between Langmuir and BET,[53] (iii) The lack of lateral interaction in these traditional models has been recognized as the root cause of the problem, yet introducing lateral interactions led to the complication of isotherm models.[41] Note that (ii) can be overcome by the generalization of the BET isotherm by Gritti and Guiochon,[59] even though their parameters remain phenomenological (see Section B.6). In contrast, the ABC isotherm captures Types I, II, III effortlessly as the spectrum of sorbate-sorbate repulsion to attraction.[41,54]

B.5 Sorption Above the Critical Temperature

Sorption isotherms above the critical temperature pose difficulties for traditional models, such as the BET model. The BET model assumes that the energy of adsorption for the

second layer onward is equal to the heat of liquefaction.[53] As a consequence, the linearized BET plot cannot be produced above the critical temperature, because saturation pressure cannot be defined. In contrast, our theoretical formulation via $\rho_1 = P_1/RT$ is free from such difficulties.

B.6 Extension to Liquid Isotherm

Our sorption theory can readily be generalized to sorption from solution by exploiting (i) the analogy between gas-gas interaction and solute-solute potential of mean force and (ii) the ensemble transformation.[61] Here, we only briefly summarize its essentials, leaving the details to our recent papers.[60,61,76] The key to the generalization is twofold: (i) generalizing G_{11} in eq B.6 to $G_{22} + G_{11} - 2G_{12}$ via the sorbate–sorbate (G_{22}), solvent–solvent (G_{11}), and solvent–sorbate (G_{12}) Kirkwood–Buff integrals. (ii) The solvent–sorbate exchange coefficient $K_e = \langle n_2^* \rangle \langle n_1^r \rangle / (\langle n_1^* \rangle \langle n_2^r \rangle)$ (Eq. 7c of Heamen et al.[60]). These quantities will be employed to capture solution structure and applied to quantify the bulk-interface solution structure difference for solution isotherms.[76] Through this, the phenomenological constants adopted previously (for example, the extended BET model, which has been used for chromatographic isotherms, whose parameters are related only to "the strength of the solute–solute interactions in the condensed state" yet has "nothing to do with the adsorbent surface properties"[59]) can be attributed a full physical interpretation, without any need for assuming weak solvent adsorption or bulk-like outer-layer behavior,[59] via the Kirkwood-Buff integrals.[60]

B.7 Temperature Dependence of Sorption

How sorption depends on temperature plays an important role in the isotherm theory. However, the classical isotherm models (such as Langmuir, BET, and GAB) incorporated temperature dependence of the parameters in a model-specific manner, or even different implementation of temperature dependence coexists within the same model (see the Introduction section of Ref [77] for a review). A more general approach is based on isosteric thermo-

dynamic quantities via van't Hoff-like analysis, of which the characteristic curve on the adsorption energy representation is its special case.[77]

Here, we provide a brief summary of our theory on the temperature dependence of sorption, which has synthesized (i) the statistical thermodynamic fluctuation theory and (ii) the isosteric thermodynamics of sorption.[77] When plotted against the conventional adsorption potential $\epsilon = -RT \ln a_2$, the temperature dependence of the isotherm collapses and becomes temperature-independent, only when the sorbate number increment contributes purely energetically to the interface; otherwise, its temperature dependence reflects a correlation between sorbate number and entropy.[77] Such an insight into fluctuation is extractable from conventional temperature-dependent fitted models (e.g., the GAB model with van't Hoff-style parametrization; see eqs. 37-40 and Fig. 4 of Ref [77]). When a_2 is not definable, the adoption of P_2 (gas pressure) can yield the energetic and entropic insights (see Appendix A of Ref [77]).

C Sorption of Gas Mixtures

C.1 Isotherm Generating Equation for Binary Sorbate Mixtures

Here, we generalize the isotherm generating equation to multicomponent sorbate mixtures. Our first step is to relate the total differential $d \ln\langle n_i \rangle$ to those of the sorbate activities. For simplicity, we will focus on binary sorbate mixtures; the method presented here can be generalized straightforwardly to any number of sorbate components. In the following, we will present the derivation for $\langle n_1 \rangle$, from which the expression for $\langle n_2 \rangle$ can be obtained simply by swapping the indexes.

The first step is to recognize $\ln\langle n_1 \rangle$ as a function of μ_1 and μ_2, which leads to

$$d \ln\langle n_1 \rangle = \left(\frac{\partial \ln\langle n_1 \rangle}{\partial \mu_1} \right)_{\mu_2} d\mu_1 + \left(\frac{\partial \ln\langle n_1 \rangle}{\partial \mu_2} \right)_{\mu_1} d\mu_2 \tag{C.1}$$

The partial derivatives in eq C.1 can be expressed in terms of the excess numbers, N_{ij}, using eq 5, as

$$d \ln\langle n_1\rangle = (N_{11} + 1)\, d \ln \rho_1 + N_{12}\ d \ln \rho_2 \tag{C.2}$$

which can be transformed to

$$-d \ln \frac{\rho_1}{\langle n_1\rangle} = N_{11}\ d \ln \rho_1 + N_{12}\ d \ln \rho_2 \tag{C.3}$$

From eq C.3, straightforward algebra leads to

$$-d \frac{\rho_1}{\langle n_1\rangle} = \frac{N_{11}}{\langle n_1\rangle}\ d\rho_1 + \frac{\rho_1}{\rho_2}\frac{N_{12}}{\langle n_1\rangle}\ d\rho_2 \tag{C.4}$$

which is the isotherm-generating equation for binary sorbate mixtures.

C.2 Characteristic Equations

Here, we generalize the derivation of the single-component ABC isotherm to binary sorbate mixtures. This can be achieved by introducing the Taylor expansions of $\frac{N_{11}}{\langle n_1\rangle}$ and $\frac{\rho_1}{\rho_2}\frac{N_{12}}{\langle n_1\rangle}$ around the reference point, $\boldsymbol{\rho}^r = (\rho_1^r, \rho_2^r)$, as

$$\begin{aligned} \frac{N_{11}}{\langle n_1\rangle} &= B_{11}^{\boldsymbol{\rho}^r} + C_{111}^{\boldsymbol{\rho}^r}(\rho_1 - \rho_1^r) + C_{112}^{\boldsymbol{\rho}^r}(\rho_2 - \rho_2^r) + \cdots \\ \frac{\rho_1}{\rho_2}\frac{N_{12}}{\langle n_1\rangle} &= B_{12}^{\boldsymbol{\rho}^r} + C_{121}^{\boldsymbol{\rho}^r}(\rho_1 - \rho_1^r) + C_{122}^{\boldsymbol{\rho}^r}(\rho_2 - \rho_2^r) + \cdots \end{aligned} \tag{C.5}$$

where $B_{ij}^{\boldsymbol{\rho}^r}$ and $C_{ijk}^{\boldsymbol{\rho}^r}$ are the expansion coefficients.

For $B_{11}^{\boldsymbol{\rho}^r}$ and $B_{12}^{\boldsymbol{\rho}^r}$ can be interpreted straightforwardly from eq C.5 via

$$\begin{aligned} B_{11}^{\boldsymbol{\rho}^r} &= \left(\frac{N_{11}}{\langle n_1\rangle}\right)_{\boldsymbol{\rho}=\boldsymbol{\rho}^r} = \left(\frac{G_{11}}{v}\right)_{\boldsymbol{\rho}=\boldsymbol{\rho}^r} \\ B_{12}^{\boldsymbol{\rho}^r} &= \left(\frac{\rho_1}{\rho_2}\frac{N_{12}}{\langle n_1\rangle}\right)_{\boldsymbol{\rho}=\boldsymbol{\rho}^r} = \left(\frac{\rho_1\langle n_2\rangle}{\rho_2\langle n_1\rangle}\frac{N_{12}}{\langle n_2\rangle}\right)_{\boldsymbol{\rho}=\boldsymbol{\rho}^r} = \left(\frac{A_1}{A_2}\frac{G_{12}}{v}\right)_{\boldsymbol{\rho}=\boldsymbol{\rho}^r} \end{aligned} \tag{C.6}$$

where G_{ij} is the interfacial Kirkwood-Buff integral, v is the volume of the interface. The C parameters are defined as

$$\begin{aligned}
C_{111}^{\boldsymbol{\rho}^r} &= \left(\frac{\partial}{\partial \rho_1}\frac{N_{11}}{\langle n_1\rangle}\right)_{\boldsymbol{\rho}=\boldsymbol{\rho}^r} \\
C_{112}^{\boldsymbol{\rho}^r} &= \left(\frac{\partial}{\partial \rho_2}\frac{N_{11}}{\langle n_1\rangle}\right)_{\boldsymbol{\rho}=\boldsymbol{\rho}^r} \\
C_{121}^{\boldsymbol{\rho}^r} &= \left(\frac{\partial}{\partial \rho_1}\frac{\rho_1 N_{12}}{\rho_2\langle n_1\rangle}\right)_{\boldsymbol{\rho}=\boldsymbol{\rho}^r} \\
C_{122}^{\boldsymbol{\rho}^r} &= \left(\frac{\partial}{\partial \rho_2}\frac{\rho_1 N_{12}}{\rho_2\langle n_1\rangle}\right)_{\boldsymbol{\rho}=\boldsymbol{\rho}^r}
\end{aligned} \tag{C.7}$$

The evaluation of the derivatives in eq C.7 is facilitated by (i) $\partial/\partial\rho_i = (1/\rho_i)(\partial/\partial \ln \rho_i)$ and (ii) adopting the inhomogeneous ensemble for the expressions of N_{11} and N_{12},[70] via eq 5. Through (i) and (ii), eq C.7 becomes

$$\begin{aligned}
C_{111}^{\boldsymbol{\rho}^r} &= \left(\frac{1}{\rho_1}\frac{\partial}{\partial \ln \rho_1}\frac{\langle n_1\rangle_1 - \langle n_1\rangle}{\langle n_1\rangle}\right)_{\boldsymbol{\rho}=\boldsymbol{\rho}^r} \\
C_{112}^{\boldsymbol{\rho}^r} &= \left(\frac{1}{\rho_2}\frac{\partial}{\partial \ln \rho_2}\frac{\langle n_1\rangle_1 - \langle n_1\rangle}{\langle n_1\rangle}\right)_{\boldsymbol{\rho}=\boldsymbol{\rho}^r} \\
C_{121}^{\boldsymbol{\rho}^r} &= \left(\frac{1}{\rho_1}\frac{\partial}{\partial \ln \rho_1}\frac{\rho_1\left(\langle n_2\rangle_1 - \langle n_2\rangle\right)}{\rho_2\langle n_1\rangle}\right)_{\boldsymbol{\rho}=\boldsymbol{\rho}^r} \\
C_{122}^{\boldsymbol{\rho}^r} &= \left(\frac{1}{\rho_2}\frac{\partial}{\partial \ln \rho_2}\frac{\rho_1\left(\langle n_2\rangle_1 - \langle n_2\rangle\right)}{\rho_2\langle n_1\rangle}\right)_{\boldsymbol{\rho}=\boldsymbol{\rho}^r}
\end{aligned} \tag{C.8}$$

Evaluating the derivatives of eq C.8 yields

$$\begin{aligned}
C_{111}^{\boldsymbol{\rho}^r} &= \left\{\frac{\langle n_1\rangle}{\rho_1^r}\left[\frac{(\langle\delta n_1\delta n_1\rangle_1 - \langle n_1\rangle_1) - (\langle\delta n_1\delta n_1\rangle - \langle n_1\rangle)}{\langle n_1\rangle^2} - \frac{(\langle n_1\rangle_1 - \langle n_1\rangle)^2}{\langle n_1\rangle^2}\right]\right\}_{\boldsymbol{\rho}=\boldsymbol{\rho}^r} \\
C_{112}^{\boldsymbol{\rho}^r} &= C_{121}^{\boldsymbol{\rho}^r} = \left\{\frac{\langle n_2\rangle}{\rho_2^r}\left[\frac{\langle\delta n_1\delta n_2\rangle_1 - \langle\delta n_1\delta n_2\rangle}{\langle n_1\rangle\langle n_2\rangle} - \frac{(\langle n_1\rangle_1 - \langle n_1\rangle)(\langle n_2\rangle_1 - \langle n_2\rangle)}{\langle n_1\rangle\langle n_2\rangle}\right]\right\}_{\boldsymbol{\rho}=\boldsymbol{\rho}^r} \\
C_{122}^{\boldsymbol{\rho}^r} &= \left\{\frac{\rho_1\langle n_2\rangle^2}{(\rho_2)^2\langle n_1\rangle}\left[\frac{(\langle\delta n_2\delta n_2\rangle_1 - \langle n_2\rangle_1) - (\langle\delta n_2\delta n_2\rangle - \langle n_2\rangle)}{\langle n_2\rangle^2} - \frac{(\langle n_2\rangle_1 - \langle n_2\rangle)^2}{\langle n_2\rangle^2}\right]\right\}_{\boldsymbol{\rho}=\boldsymbol{\rho}^r}
\end{aligned} \tag{C.9}$$

Using the definitions of the excess numbers (eqs 5 and 6) and their generalizations to inhomogeneous ensembles,

$$N_{k,ij} = \frac{\langle \delta n_i \delta n_j \rangle_k - \delta_{ij} \langle n_j \rangle_k}{\langle n_i \rangle_k} \tag{C.10}$$

eq C.9 can be simplified to

$$\begin{aligned}
C_{111}^{\boldsymbol{\rho}^r} &= \frac{1}{A_1^r} \left[\frac{\langle n_1 \rangle_1 N_{1,11} - \langle n_1 \rangle N_{11} - N_{11}^2}{\langle n_1 \rangle^2} \right]_{\boldsymbol{\rho}=\boldsymbol{\rho}^r} \\
C_{112}^{\boldsymbol{\rho}^r} &= C_{121}^{\boldsymbol{\rho}^r} = \frac{1}{A_2^r} \left[\frac{\langle n_1 \rangle_1 N_{1,12} - \langle n_1 \rangle N_{12} - N_{11} N_{12}}{\langle n_1 \rangle \langle n_2 \rangle} \right]_{\boldsymbol{\rho}=\boldsymbol{\rho}^r} \\
C_{122}^{\boldsymbol{\rho}^r} &= \frac{A_1^r}{(A_2^r)^2} \left[\frac{\langle n_2 \rangle_1 N_{1,22} - \langle n_2 \rangle N_{22} - N_{12}^2}{\langle n_2 \rangle^2} \right]_{\boldsymbol{\rho}=\boldsymbol{\rho}^r}
\end{aligned} \tag{C.11}$$

C.3 Multicomponent ABC Isotherm

C.3.1 General Expression

Here, we generalize the ABC isotherm, previously derived for single-component sorbates,[53,54,65] to multicomponent sorbate mixtures. This can be achieved by solving the differential equation, as a combination of the isotherm-generating equation (eq C.4) and the characteristic equation (eq C.5), which yields

$$\begin{aligned}
\frac{\rho_1}{\langle n_1 \rangle} &= A_1^{\boldsymbol{\rho}^r} - B_{11}^{\boldsymbol{\rho}^r}(\rho_1 - \rho_1^r) - B_{12}^{\boldsymbol{\rho}^r}(\rho_2 - \rho_2^r) \\
&\quad - \frac{C_{111}^{\boldsymbol{\rho}^r}}{2}(\rho_1 - \rho_1^r)^2 - \frac{C_{122}^{\boldsymbol{\rho}^r}}{2}(\rho_2 - \rho_2^r)^2 - C_{112}^{\boldsymbol{\rho}^r}(\rho_1 - \rho_1^r)(\rho_2 - \rho_2^r)
\end{aligned} \tag{C.12}$$

and by symmetry

$$\begin{aligned}
\frac{\rho_2}{\langle n_2 \rangle} &= A_2^{\boldsymbol{\rho}^r} - B_{21}^{\boldsymbol{\rho}^r}(\rho_1 - \rho_1^r) - B_{22}^{\boldsymbol{\rho}^r}(\rho_2 - \rho_2^r) \\
&\quad - \frac{C_{211}^{\boldsymbol{\rho}^r}}{2}(\rho_1 - \rho_1^r)^2 - \frac{C_{222}^{\boldsymbol{\rho}^r}}{2}(\rho_2 - \rho_2^r)^2 - C_{221}^{\boldsymbol{\rho}^r}(\rho_1 - \rho_1^r)(\rho_2 - \rho_2^r)
\end{aligned} \tag{C.13}$$

Thus, eqs C.12 and C.13 constitute the solution of the total differentiatial equation. The ABC isotherm can readily be obtained by rewriting eqs C.12 and C.13 in the form of $\langle n_1 \rangle(\rho_1, \rho_2)$ and $\langle n_2 \rangle(\rho_1, \rho_2)$. In the following, we will present the two special cases that are particularly useful.

C.3.2 Global Binary ABC Isotherm

As the generalization of the ABC isotherm for single sorbates (eq B.11), here we present the multicomponent ABC isotherm expanded around $\boldsymbol{\rho} = (0, 0)$, which will be referred to as the global multicomponent ABC isotherm. From eqs C.12 and C.13, we obtain

$$\begin{aligned} \langle n_1 \rangle &= \frac{\rho_1}{A_1^0 - B_{11}^0 \rho_1 - B_{12}^0 \rho_2 - \frac{C_{111}^0}{2}\rho_1^2 - \frac{C_{122}^0}{2}\rho_2^2 - C_{112}^0 \rho_1 \rho_2} \\ \langle n_2 \rangle &= \frac{\rho_2}{A_2^0 - B_{22}^0 \rho_2 - B_{21}^0 \rho_1 - \frac{C_{222}^0}{2}\rho_2^2 - \frac{C_{211}^0}{2}\rho_1^2 - C_{221}^0 \rho_1 \rho_2} \end{aligned} \tag{C.14}$$

where the superscript 0 for the isotherm parameters signifies $\boldsymbol{\rho} = (0, 0)$ as the reference. Note that the definition of B_{12} and B_{21} (eq C.6), alongside the symmetry $G_{12} = G_{21}$, leads to the following relationship between B_{12} and B_{21}.

$$B_{21}^0 = \left(\frac{A_2^0}{A_1^0} \right)^2 B_{12}^0 \tag{C.15}$$

C.3.3 Global Binary AB Isotherm as a Generalization of the Extended Langmuir Model

Here, we derive the global binary AB isotherm as a special case of the corresponding ABC isotherm (C.14), as

$$\begin{aligned} \langle n_1 \rangle &= \frac{\rho_1}{A_1^0 - B_{11}^0 \rho_1 - B_{12}^0 \rho_2} \\ \langle n_2 \rangle &= \frac{\rho_2}{A_2^0 - B_{22}^0 \rho_2 - B_{21}^0 \rho_1} \end{aligned} \tag{C.16}$$

When using the AB isotherm (eq C.16), the constraint between B_{12} and B_{21} via eq C.15 must be taken into account.

D Isotherms Along a Constant Total Pressure

D.1 Isotherm Generating Relationship and Characteristic Equation

Our goal is to derive a binary sorption isotherm along a constant total pressure, $P = P_1 + P_2 =$ const. This is equivalent to $\rho = \rho_1 + \rho_2 =$ const. Let us first focus on the isotherm generating relationship for $\langle n_1 \rangle$. Adopting $\boldsymbol{\rho}^r = (\rho, 0)$ as a reference point, and consider how $\langle n_1 \rangle$ changes as we increase the mole fraction of sorbate species 2 in the gas phase. To this end, we take y_2 as the sole variable, and can express

$$\begin{aligned} \rho_1 - \rho_1^r &= -\rho y_2 \\ \rho_2 - \rho_2^r &= \rho y_2 \end{aligned} \tag{D.1}$$

The differentials $d\rho_1$ and $d\rho_2$ can be expressed as

$$d\rho_1 = \rho dy_1 = -\rho dy_2, \qquad d\rho_2 = -\rho dy_1 = \rho dy_2 \tag{D.2}$$

With this setup, the isotherm generating relationship (eq C.4) can be expressed as

$$d\frac{\rho_1}{\langle n_1 \rangle} = \rho \left(\frac{N_{11}}{\langle n_1 \rangle} - \frac{\rho_1}{\rho_2} \frac{N_{12}}{\langle n_1 \rangle} \right) dy_2 \tag{D.3}$$

Now, we express the characteristic equation for the ABC isotherm (eq C.5) using y_2, as

$$\begin{aligned}\frac{N_{11}}{\langle n_1\rangle} &= B_{11}^{(\rho,0)} + \rho\left(C_{112}^{(\rho,0)} - C_{111}^{(\rho,0)}\right) y_2 + \cdots \\ \frac{\rho_1}{\rho_2}\frac{N_{12}}{\langle n_1\rangle} &= B_{12}^{(\rho,0)} + \rho\left(C_{122}^{(\rho,0)} - C_{121}^{(\rho,0)}\right) y_2 + \cdots\end{aligned} \tag{D.4}$$

Note that (i) $C_{112}^{(\rho,0)}$ and $C_{122}^{(\rho,0)}$ express the ρ_2-derivatives of $N_{11}/\langle n_1\rangle$ and $N_{12}/\langle n_1\rangle$ and (ii) $C_{111}^{(\rho,0)}$ and $C_{121}^{(\rho,0)}$ express the ρ_1-derivatives. Thus, combining eqs D.3 and D.4 yields the differential equation for the ABC isotherm,

$$d\frac{\rho_1}{\langle n_1\rangle} = \left(\rho\Delta B_1^{(\rho,0)} - \rho^2\Delta C_1^{(\rho,0)} y_2\right) dy_2 \tag{D.5}$$

where

$$\begin{aligned}\Delta B_1^{(\rho,0)} &\equiv B_{11}^{(\rho,0)} - B_{12}^{(\rho,0)} \\ \Delta C_1^{(\rho,0)} &\equiv C_{111}^{(\rho,0)} - 2C_{112}^{(\rho,0)} + C_{122}^{(\rho,0)}\end{aligned} \tag{D.6}$$

are the preferential and conditional self-interactions defined at $\boldsymbol{\rho}^r = (\rho, 0)$. By symmetry, the combined differential equation for $\langle n_2\rangle$ can be expressed as

$$d\frac{\rho_2}{\langle n_2\rangle} = \left(\rho\Delta B_2^{(0,\rho)} - \rho^2\Delta C_2^{(0,\rho)} y_1\right) dy_1 \tag{D.7}$$

where

$$\begin{aligned}\Delta B_2^{(0,\rho)} &\equiv B_{22}^{(0,\rho)} - B_{21}^{(0,\rho)} \\ \Delta C_2^{(0,\rho)} &\equiv C_{222}^{(0,\rho)} - 2C_{221}^{(0,\rho)} + C_{211}^{(0,\rho)}\end{aligned} \tag{D.8}$$

are the preferential and conditional self-interactions defined at $\boldsymbol{\rho}^r = (0, \rho)$. We will return to the physical meanings of ΔB_i and ΔC_i once we have derived the isotherm.

D.2 The ABC Isotherm

Using eq D.1, eq D.5 becomes

$$\langle n_1 \rangle = \frac{\rho y_1}{A_1^{(\rho,0)} + \Delta B_1^{(\rho,0)} \rho y_2 - \frac{1}{2} \Delta C_1^{(\rho,0)} \rho^2 y_2^2} \tag{D.9}$$

where preferential and conditional self-interaction parameters, $\Delta B_1^{(\rho,0)}$ and $\Delta C_1^{(\rho,0)}$ are defined by eq D.6. By symmetry,

$$\langle n_2 \rangle = \frac{\rho y_2}{A_2^{(0,\rho)} + \Delta B_2^{(0,\rho)} \rho y_1 - \frac{1}{2} \Delta C_2^{(0,\rho)} \rho^2 y_1^2} \tag{D.10}$$

where the interpretation of the parameters are described in the main text.

D.3 ΔC as Conditional χ

Here, we clarify the physical meaning of $\Delta C_1^{(\rho,0)}$ (eq D.6) and $\Delta C_2^{(0,\rho)}$ (eq D.8). We will focus on interpreting $\Delta C_1^{(\rho,0)}$, from which $\Delta C_2^{(0,\rho)}$ can be interpreted through symmetry. Through simple algebra, $\Delta C_1^{(\rho,0)}$ can be expressed as

$$\Delta C_1^{(\rho,0)} = C_{111}^{(\rho,0)} - 2C_{112}^{(\rho,0)} + C_{122}^{(\rho,0)} = \frac{\chi_1^{(\rho,0)}}{A_1^{(\rho,0)}} - \frac{\left(\Delta B_1^{(\rho,0}\right)^2}{A_1^{(\rho,0)}} \tag{D.11}$$

where

$$\chi_1^{(\rho,0)} \equiv \left[\frac{\langle n_1 \rangle_1 N_{1,11} - \langle n_1 \rangle N_{11}}{\langle n_1 \rangle^2} - \frac{2A_1}{A_2} \frac{\langle n_1 \rangle_1 N_{1,12} - \langle n_1 \rangle N_{12}}{\langle n_1 \rangle \langle n_2 \rangle} + \frac{A_1^2}{A_2^2} \frac{\langle n_2 \rangle_1 N_{1,22} - \langle n_2 \rangle N_{22}}{\langle n_2 \rangle^2} \right]_{\boldsymbol{\rho}=(\rho,0)} \tag{D.12}$$

is the net self-interaction (self minus mutual) in the presence of a probe sorbate of species 1. Likewise, by symmetry,

$$\Delta C_2^{(0,\rho)} = C_{222}^{(0,\rho)} - 2C_{221}^{(0,\rho)} + C_{211}^{(0,\rho)} = \frac{\chi_2^{(0,\rho)}}{A_2^{(0,\rho)}} - \frac{\left(\Delta B_2^{(0,\rho)}\right)^2}{A_2^{(0,\rho)}} \tag{D.13}$$

where

$$\chi_2^{(0,\rho)} \equiv \left[\frac{\langle n_2\rangle_2 N_{2,22} - \langle n_2\rangle N_{22}}{\langle n_2\rangle^2} - \frac{2A_2}{A_1}\frac{\langle n_2\rangle_2 N_{2,21} - \langle n_2\rangle N_{21}}{\langle n_2\rangle\langle n_1\rangle} + \frac{A_2^2}{A_1^2}\frac{\langle n_1\rangle_2 N_{2,11} - \langle n_1\rangle N_{11}}{\langle n_1\rangle^2}\right]_{\boldsymbol{\rho}=(0,\rho)} \tag{D.14}$$

With the introduction of the selectivity constant via eq 7, we obtain eqs 33 and 34 of the main text.

D.4 Partitioning versus Exchange

What distinguishes the AB isotherm from the Langmuirian models is its focus on sorbate-sorbate KBIs. We have shown previously for pure sorbates that the "Langmuir constant", K_L can be reinterpreted via the AB isotherm as $K_L = -B_{11}^0/A_1^0$.[41,54] Its generalization to selective isotherm can be obtained via a straightforward combination of eqs 7, 24, 30, and 32:

$$\begin{aligned} \frac{\Delta B_1^{(\rho,0)}}{A_1^{(\rho,0)}} &= (K_1G_{11} - K_2G_{12})^{(\rho,0)} \\ \frac{\Delta B_2^{(0,\rho)}}{A_2^{(0,\rho)}} &= (K_2G_{22} - K_1G_{21})^{(0,\rho)} \end{aligned} \tag{D.15}$$

Equation D.15 has a clear interpretation as the preferential self-interaction (i.e., between gas species i and the probe species i; Figure S1). However, when applied to interpret experimental data (Table S1), $\Delta B/A$ does not exhibit a clear correspondence either to isotherm shapes (Figure 8) or to the interfacial mole fraction plot (Figure 9). This is in

contrast to the clarity of the AB parameters in Table 4 for elucidating the difference between the sorbate pairs.

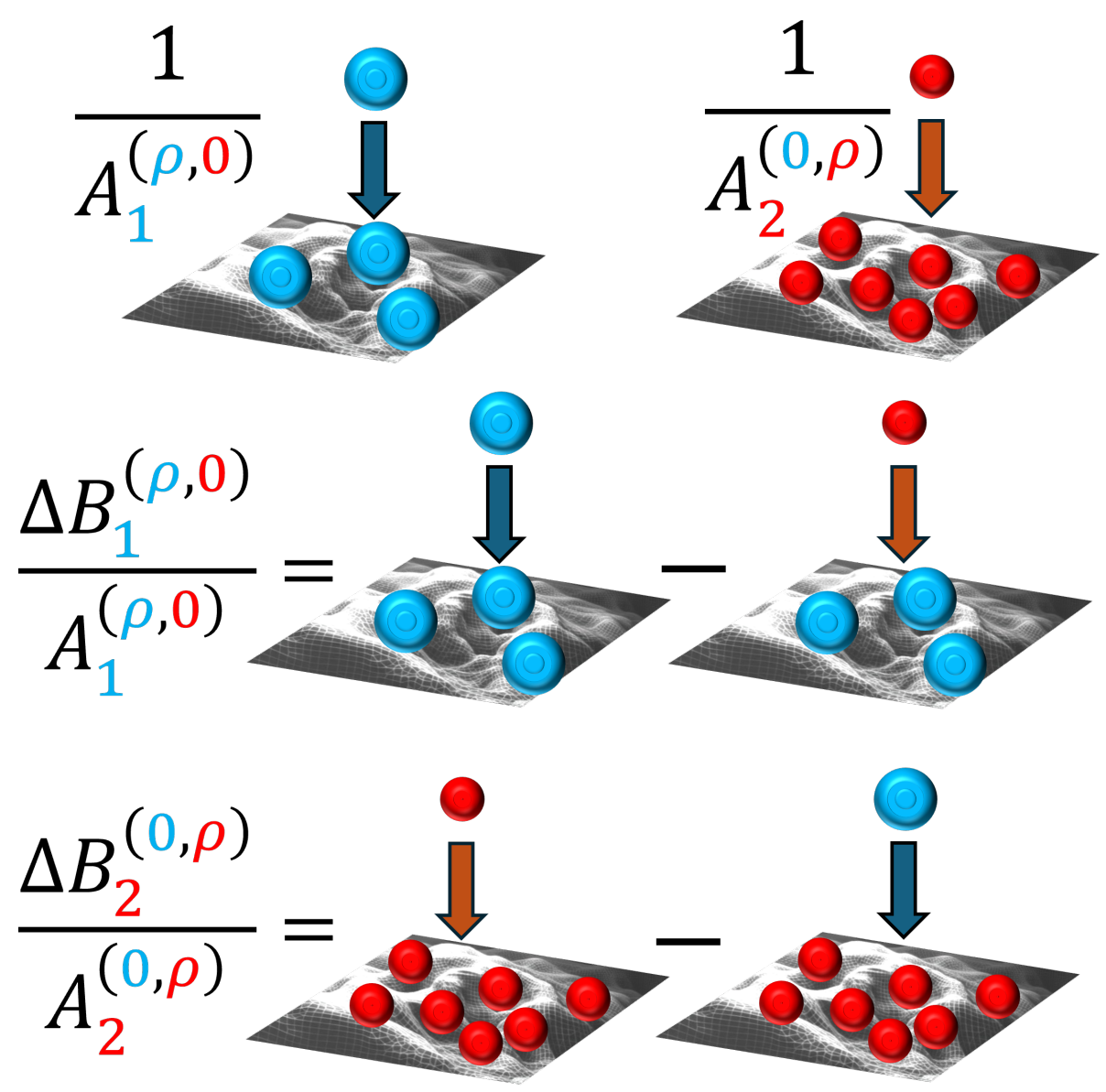

Figure S1: A schematic diagram for the parameters of the ABC isotherm. $1/A_1^{(\rho,0)}$ and $1/A_2^{(0,\rho)}$ signify (via eq 30) the sorbate-surface interaction at $(\rho_1, \rho_2) = (\rho, 0)$ and $(0, \rho)$, respectively. $\Delta B_1^{(\rho,0)}/A_1^{(\rho,0)}$ and $\Delta B_2^{(0,\rho)}/A_2^{(0,\rho)}$ (that have a more straightforward interpretation than ΔB_i) signify (via eq D.15) the preferential self-interaction at the interface.

Table S1: Selective Sorption via Eq D.15

sorbate (1)	sorbate (2)	$1/A_1^{(\rho,0)\text{a}}$	$1/A_2^{(0,\rho)\text{a}}$	$\Delta B_1^{(\rho,0)}/A_1^{(\rho,0)\text{a}}$	$\Delta B_2^{(0,\rho)}/A_2^{(0,\rho)\text{a}}$
ethylene	ethane	0.106	0.115	0.0692	-0.1251
ethane	propylene	0.115	0.348	0.5350	-0.1634
ethylene	propylene	0.106	0.348	1.3771	-0.1812
methane	ethylene	0.0150	0.106	0.2590	-0.1483
methane	ethane	0.0150	0.115	1.1542	-0.1378

[a]Calculated from Table 4.

E Understanding Binary Sorption Isotherm Shapes

E.1 Analogy to Single-Component Isotherms

Here, we show how we can elucidate the shapes of $\langle n_1 \rangle$ and $\langle n_2 \rangle$ for binary isotherms using the analogy with single-component sorption isotherms. This can be achieved by rewriting the binary ABC isotherm along constant ρ (eq 29) into the following form:

$$\langle n_i \rangle = \frac{\rho_i}{\tilde{A}_i - \tilde{B}_i \rho_i - \frac{1}{2}\tilde{C}_i \rho_i^2} \tag{E.1}$$

Comparing eqs 29 and E.1 yields the following correspondence:

$$\begin{aligned}
\tilde{A}_1 &= A_1^{(\rho,0)} + \rho \Delta B_1^{(\rho,0)} - \frac{1}{2}\rho^2 \Delta C_1^{(\rho,0)}, \\
\tilde{A}_2 &= A_2^{(0,\rho)} + \rho \Delta B_2^{(0,\rho)} - \frac{1}{2}\rho^2 \Delta C_2^{(0,\rho)}, \\
\tilde{B}_1 &= \Delta B_1^{(\rho,0)} - \rho \Delta C_1^{(\rho,0)}, \quad \tilde{B}_2 = \Delta B_2^{(0,\rho)} - \rho \Delta C_2^{(0,\rho)} \\
\tilde{C}_1 &= \Delta C_1^{(\rho,0)}, \quad \tilde{C}_2 = \Delta C_2^{(0,\rho)}
\end{aligned} \tag{E.2}$$

which will be employed in the main text to explain the functional shapes.

E.2 Analogy to the Extended Langmuir Model

Here, we establish a mathematical analogy between our binary ABC isotherm (along a constant pressure, eq 29) and the Extended Langmuir model, which has the general form

$$\begin{aligned}
\langle n_1 \rangle &= \frac{n_1^{(L)} K_{11}^{(L)} \rho_1}{1 + K_{11}^{(L)} \rho_1 + K_{12}^{(L)} \rho_2} \\
\langle n_2 \rangle &= \frac{n_2^{(L)} K_{22}^{(L)} \rho_2}{1 + K_{21}^{(L)} \rho_1 + K_{22}^{(L)} \rho_2}
\end{aligned} \tag{E.3}$$

where $n_1^{(L)}$ and $n_2^{(L)}$ are the "saturation capacities" of species 1 and 2, and $K_{ij}^{(L)}$ are the "Langmuir constants", respectively. Our goal is to rewrite our binary AB isotherm (eq 29

with $\Delta C_i = 0$) along constant ρ in the mathematically analogous format to eq E.3. This can be achieved by rewriting the denominators of eq 29 as

$$\begin{aligned} A_1^{(\rho,0)} + \Delta B_1^{(\rho,0)} \rho_2 &= \left(A_1^{(\rho,0)} + B_{11}^{(\rho,0)} \rho \right) - B_{11}^{(\rho,0)} \rho_1 - B_{12}^{(\rho,0)} \rho_2 \\ A_2^{(0,\rho)} + \Delta B_2^{(0,\rho)} \rho_1 &= \left(A_2^{(0,\rho)} + B_{22}^{(0,\rho)} \rho \right) - B_{21}^{(0,\rho)} \rho_1 - B_{22}^{(0,\rho)} \rho_2 \end{aligned} \tag{E.4}$$

Here, we note that $A_1^{(0,0)}$ and $A_2^{(0,0)}$ can be obtained using from the ABC isotherms defined at $(\rho, 0)$ and $(0, \rho)$, as

$$\begin{aligned} \frac{1}{A_1^{(0,0)}} \equiv \left(\frac{\langle n_1 \rangle}{\rho_1} \right)_{(0,0)} &= \frac{1}{A_1^{(\rho,0)} - B_{11}^{(\rho,0)} (0 - \rho)} \\ \frac{1}{A_2^{(0,0)}} \equiv \left(\frac{\langle n_2 \rangle}{\rho_2} \right)_{(0,0)} &= \frac{1}{A_2^{(0,\rho)} - B_{22}^{(0,\rho)} (0 - \rho)} \end{aligned} \tag{E.5}$$

Substituting eqs E.4 and E.5 into eq 29 yields eq 36 of the main text.

Comparing eq 36 with the Extended Langmuir (eq 35), we obtain

$$\begin{aligned} n_1^{(L)} &= -\frac{1}{B_{11}^{(\rho,0)}}, \quad n_2^{(L)} = -\frac{1}{B_{22}^{(0,\rho)}} \\ K_{11}^{(L)} &= -\frac{B_{11}^{(\rho,0)}}{A_1^{(0,0)}}, \quad K_{12}^{(L)} = -\frac{B_{12}^{(\rho,0)}}{A_1^{(0,0)}} \\ K_{22}^{(L)} &= -\frac{B_{22}^{(0,\rho)}}{A_2^{(0,0)}}, \quad K_{21}^{(L)} = -\frac{B_{21}^{(0,\rho)}}{A_2^{(0,0)}} \end{aligned} \tag{E.6}$$

To simplify eq E.6, we note that $C_{111} = C_{222} = 0$ for pure sorption isotherm. Consequently, B_{11} and B_{22} for pure sorbates are independent of ρ, hence

$$B_{11}^{(\rho,0)} = B_{11}^{(0,0)}, \quad B_{22}^{(0,\rho)} = B_{22}^{(0,0)} \tag{E.7}$$

We also use the above, in combination with the definitions of $\Delta B_1^{(\rho,0)}$ and $\Delta B_2^{(0,\rho)}$ (eq 28),

to obtain

$$
\begin{aligned}
B_{12}^{(\rho,0)} &= B_{11}^{(\rho,0)} - \Delta B_1^{(\rho,0)} \\
B_{21}^{(0,\rho)} &= B_{22}^{(0,\rho)} - \Delta B_2^{(0,\rho)}
\end{aligned} \tag{E.8}
$$

Substituting eqs E.7 and E.8 into eq E.6, we obtain eq 37 of the main text.

F Goodness of Fit (R^2) Values for the ABC and AB Isotherms

Here, we summarize the coefficients of determination (R^2) for the AB and ABC isotherm fits presented in the main text.

First, the AB isotherm for pure sorbates (eq 18 with $C_{111}^{(0,0)} = 0$) can successfully fit the experimental sorption isotherms (Table 2 and Figure 7), as has been evidenced by the R^2 values summarized in Table S2:

Table S2: R^2 for the AB Isotherm Fit to Pure Gas Adsorption Data on Activated Carbon

Sorbate	R^2
Methane	0.9995
Ethane	0.9922
Ethylene	0.9908
Propylene	0.9767

Second, the ABC isotherm for binary mixtures (eq 29) can provide accurate fitting for the binary sorption isotherms at constant total pressure (Table 3 and Figure 8), as evidenced by Table S3. For most of the binary sorbate pairs, the AB isotherm (i.e., eq 29 with $\Delta C_1^{(\rho,0)} = \Delta C_2^{(0,\rho)} = 0$) provides reasonable fitting, except for the methane (1)-ethane (2) pair.

Table S3: R^2 for the binary ABC and AB isotherms fit to mixed gas adsorption data on activated carbon at $\rho = 3.72$ mol m^{-3}.

Sorbate (1)	Sorbate (2)	R^2 (ABC)	R^2 (AB)
Ethylene	Ethane	0.9907	0.9630
Ethane	Propylene	0.9941	0.9732
Ethylene	Propylene	0.9900	0.9670
Methane	Ethylene	0.9990	0.9989
Methane	Ethane	0.9864	0.8939
